# Insight into Iron(III)-Tannate Biosorbent for Adsorption Desalination and Tertiary Treatment of Water Resources

**DOI:** 10.1021/acsomega.4c05152

**Published:** 2024-12-19

**Authors:** Kelvin Adrah, Gayani Pathiraja, Hemali Rathnayake

**Affiliations:** Department of Nanoscience, Joint School of Nanoscience & Nanoengineering, University of North Carolina at Greensboro, 1907 East Gate City Blvd, Greensboro, North Carolina 27401, United States

## Abstract

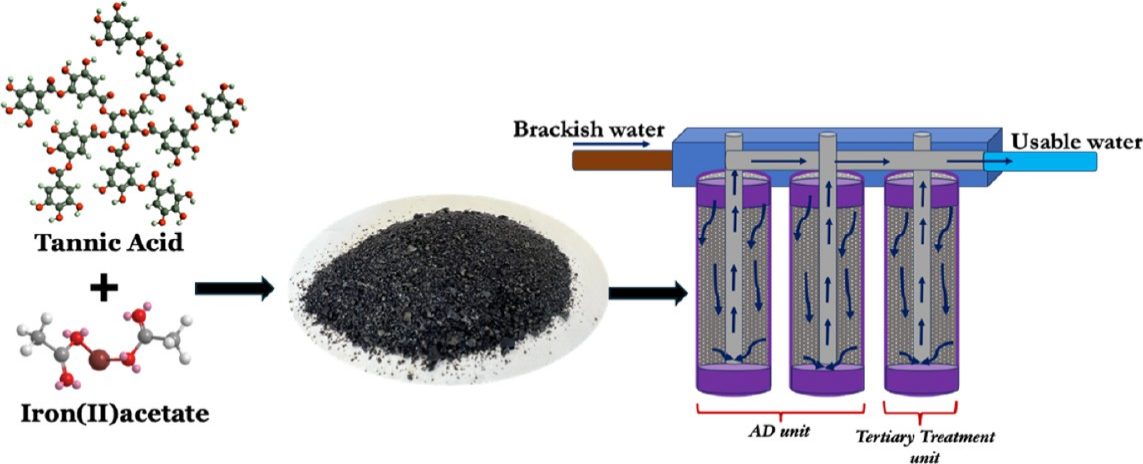

An innovative biosorbent-based water remediation unit could reduce the demand for freshwater while protecting the surface and groundwater sources by using saline water resources, such as brine, brackish water, and seawater for irrigation. Herein, for the first time, we introduce a simple, rapid, and cost-effective iron(III)-tannate biosorbent-based technology, which functions as a stand-alone fixed-bed filter system for the treatment of salinity, heavy-metal contaminants, and pathogens present in a variety of water resources. Our approach presents a streamlined, cost-efficient, energy-saving, and sustainable avenue for water treatment, distinct from current adsorption desalination or conventional membrane techniques supplemented with chemical and UV treatments for disinfection. The proof of feasibility for effective treatment of heavy metals, adsorption desalination, and cleansing of pathogens is demonstrated using synthetic water, brine, and field-collected seawater. The adsorption equilibrium and adsorption kinetic isotherm models, and mass transfer diffusion models confirmed the sorbent’s function for sieving heavy-metal ions—silver (Ag^+^), cadmium (Cd^2+^), and lead (Pb^2+^)—from water. The maximum adsorption capacities (*q*_m_) of the sorbent for Ag^+^, Cd^2+^, and Pb^2+^ reach 96.25, 66.54, and 133.83 mg/g at neutral pH. The sorbent’s affinity for heavy-metal-ion adsorption significantly increased, yielding *q*_m_ of 116.57 mg/g for Ag^+^, 104.04 mg/g for Cd^2+^, and 165.66 mg/g for Pb^2+^, at pH 9, respectively, due to the sorbent’s amphoteric nature. The pristine sorbents exhibit exceptional adsorption desalination efficacy (>70%) for removing salinity from brine and seawater, promoting heterogeneous adsorption. Fe(III)-TA’s ability to disinfect seawater, with 67% efficacy over a very short contact time (∼15 min), confirms its remarkable antimicrobial properties for contact active mode pathogens cleansing. By preventing the release of salts, heavy-metal contaminants, and pathogens into the environment, our results proved that this novel multiplex biobased sorbent approach directly contributes to the water quality of surface and groundwater resources.

## 1 Introduction

Nanotechnology offers new opportunities for traditional engineering processes for advancing existing water and wastewater technologies. Agriculture consumes more than 85% of the available freshwater.^1^ As climate change progresses, population grows, and industrial activities expand, the issue of freshwater scarcity becomes even more acute. Consequently, interest in remediating saline water sources is on the rise. Apart from freshwater and seawater, water with different levels of salinity can be found as surface and groundwater, commonly distinguished as brine and brackish water. These water resources contain a range of contaminants including heavy metals, inorganic dissolved salts, and microbial species. The wide distribution of salinity water in water-scarce regions presents an enticing alternative to traditional freshwater sources. However, finding the right balance between the chosen feedwater technology and the treatment method is crucial for enhancing energy efficiency throughout the remediation process, as the composition of saline water resources can vary with respect to the geographical region.

Currently, the percentage of brine as feedwater resource accounts typically around 60% of the volume of globally produced desalinated water.^2^ Thermal or membrane technologies are broadly considered as existing desalination processes.^3^ Seawater desalination mostly relies on thermal processes, although they are significantly energy-intensive. In general, membrane processes are suitable for the treatment of low-salinity water^3,4^ and mostly applicable in areas with substantially low energy costs.^3,4^ Membrane processes used for saltwater treatment are either pressure-driven processes or electro-driven processes. Reverse osmosis (RO) and nanofiltration (NF) are the most common pressure-driven methods and electrodialysis (ED) and (membrane) capacitive deionization (CDI) are popular as electro-driven processes.^5^ The salinity and chemical composition of water usually determine the desalination efficiency of the membrane processes. The RO provides an advantage in terms of energy efficiency for small-scale desalination plants with lower-salinity water.^6,7^ In contrast, NF is suitable for selective multivalent ion removal,^8,9^ thereby providing its utility for water softening. Despite advancements in each of these desalination techniques, substantial energy demands persist and supplementary treatment methods are required to eliminate heavy metals and microbial contaminants from these surface and groundwater resources. Moreover, membrane fouling and scaling pose significant challenges, leading to decreased freshwater production and increased energy consumption.

Adsorption desalination (AD) has emerged as an innovative technology with more environmental sustainability than conventional membrane approaches. This results in decreased electricity consumption and greenhouse gas emissions.^10^ Indeed, such methods cut down the energy consumption by almost one-third compared to that of membrane methods and operate at low-temperature conditions that can be driven by solar energy and waste heat.^10^ Furthermore, adsorption desalination systems efficiently desalinate high-salinity brine to produce high-quality, potable water. This makes them a suitable partner for integration with conventional RO systems to tackle brine disposal challenges and decrease specific energy consumption.^10^ However, adsorption desalination is still in the early stage of development, compared to thermal methods and membrane technologies.^10^ Nevertheless, current desalination techniques also require complementary systems and practices for the removal of pathogens and other chemical contaminants. Following desalination, tertiary water disinfection is usually achieved through various methods, such as chlorine, ultraviolet light, or ozone treatment. Although chlorine treatment has been widely used in water disinfection, research has demonstrated its adverse effects on aquatic systems.^11^ Additionally, the reaction between chlorine and organic matter can generate potentially carcinogenic disinfection byproducts (DBPs).^12,13^

Among the current state-of-the-art heavy-metal removal technologies, membrane-based separation technologies are widely used to replace conventional chemical-based heavy-metal treatment systems.^14^ In particular, inorganic ceramic membranes have been attractive for water remediation owing to their high thermal and chemical stability in harsh environments.^15^ The membrane porosity determines the separation process through the size exclusion mechanism. In membrane filters, high porosity and uniform pores certainly yield high permeability. However, their selectivity can be reduced.^16^ Membrane fouling is another common issue, that reduces the filtration efficiency and the lifetime of the membrane, increasing the overall cost, frequent maintenance, and membrane replacement.^17^ Instead, utilization of sorbents for heavy-metal remediation is mostly cost-effective and rather simple in terms of maintenance and operation. In general, the most widely used adsorbent for heavy-metal removal is activated carbon. Additionally, carbon nanotubes (CNTs),^18−22^ metallic nanoparticles,^23,24^ and metal oxides^25−30^ have been explored as adsorbents. Substituting activated carbon, these sorbents effectively remove both organic and metal contaminants. Their surface structures can be manipulated to maximize active adsorption sites.^25−27^ Metal oxide nanoparticles have been also incorporated onto the activated carbon surface or other porous materials to remove arsenic and organic contaminants. These approaches enable the fabrication of point-of-use devices.^28,29^ However, most known sorbents are specific to contaminant types and water chemistry, limiting their wide applicability as an effective discrete unit for both the desalination and tertiary treatment of heavy metals and pathogens.

A stand-alone water remediation unit could offer a simpler, more cost-effective, less energy-intensive, and sustainable approach to traditional multistep treatment methods as well as for overcoming their challenges in treating salinated surface and groundwater. Thus, herein, for the first time, we demonstrate adsorption desalination combined with treatment for heavy metals and pathogen removal, employing a novel multiplex biosorbent. A porous iron(III)-tannate sorbent (Fe(III)-TA), as the first bioinspired coordination polymer with a rigid framework, was synthesized from a polytopic natural polyphenol. We have previously demonstrated the use of biomass building blocks to construct this novel bioinspired adsorbent, using a green synthesis in a large scale under ambient conditions in water.^31^ The scientific significance of Fe(III)-TA as a biosorbent over current state-of-the-art natural sorbents is its multiplex function with tailorable microporosity and amphoteric surface properties for selectivity at a wider range of pH. Nonetheless, our sorbent exhibits unique physiochemical surface properties for selective interfacial interactions with a variety of impurities present in water. As demonstrated in this work, Fe(III)-TA’s ability to cleanse pathogens, salts, and heavy-metal contaminants from surface and groundwater sources before discharging into the environment highlights its novelty and potential utility to use as a self-contained unit for the selective extraction, separation, and recovery of alkali and alkaline cations, anions, toxic heavy metals, and valuable minerals from a variety of water resources.^31,32^

Demonstrating the proof-of-feasibility to use our biobased Fe(III)-tannate sorbent as a platform technology for water remediation, this study focuses on an in-depth understanding of the sorbent’s adsorption mechanism for: (1) removing lead, cadmium, and silver, (2) adsorption desalination of synthetic brines and seawater, and (3) disinfection of pathogens from seawater. Lead, cadmium, and silver often originate from industrial effluent and enter surface and groundwater resources as wastewater discharge. These are toxic metals that can be harmful to human health and the environment when present in significant quantities; lead affects the nervous system, cadmium can damage kidneys, and silver is considered a toxic element for aquatic ecosystems even at low concentrations. Thus, developing a biosorbent-based approach for the tertiary treatment of these heavy metals is crucial for the utility of nontraditional water resources. We employed the adsorption equilibrium and adsorption kinetic isotherm models, and mass transfer diffusion models, revealing the sorbent’s mechanistic pathway for heavy metals removal. Deriving a modified adsorption isotherm model that describes sigmoidal type adsorption isotherm (S_II_-type), the heavy-metal-ion adsorption onto the sorbent follows the surface adsorption via physisorption, chemisorption, and external and internal mass transfer diffusion, filling the pores. The proof-of-validation for adsorption desalination was demonstrated by studying the adsorption of alkali and alkaline cations (Na^+^, K^+^, Mg^2+^, and Ca^2+^) and deducing total salinity removal efficiency by the sorbents, using synthetic brine solutions and seawater samples. The concept of effective inactivation of pathogens present in seawater was evaluated by employing a colony formation unit assay (CFU). The results demonstrate the multiplex nature of the sorbent as a platform technology for the efficient removal of heavy metals, desalination, and disinfection of water resources with minimal energy consumption and zero carbon emission.

## 2 Results and Discussion

In our previous work, we have introduced a bioinspired adsorbent, iron(III)-tannate (Fe(III)-TA) prepared by coordinating Fe^2+^ with polytopic tannic acid, using an aqueous-based green synthesis method.^31^ As reported previously, the surface area, pore volume, and pore diameter of Fe(III)-TA were measured to be 70.47 m^2^/g, 0.44 cm^3^/g, and ∼27 nm, respectively.^31^ Like tailorable textural features of metal–organic frameworks (MOFs), the coordination polymer framework of Fe(III)-tannate offers accessibility to tailor its textural properties via structural changes to the metal node’s coordination and the polytopic organic ligand, enhancing the absorption performance and selectivity. The initial mechanistic studies conducted on its potential to be used as a sorbent for sieving lithium from brine resources have established the foundation for exploring this novel sorbent as an adsorption desalination and tertiary treatment technology for water remediation.^32^ The current study aims at providing an in-depth investigation of porous Fe(III)-TA sorbent’s multiplex capability as a stand-alone fixed-bed column system for desalination, heavy-metal remediation, and disinfection of water resources.

### 2.1 Study on Heavy-Metal-Ion Adsorption

**Figure 1:**
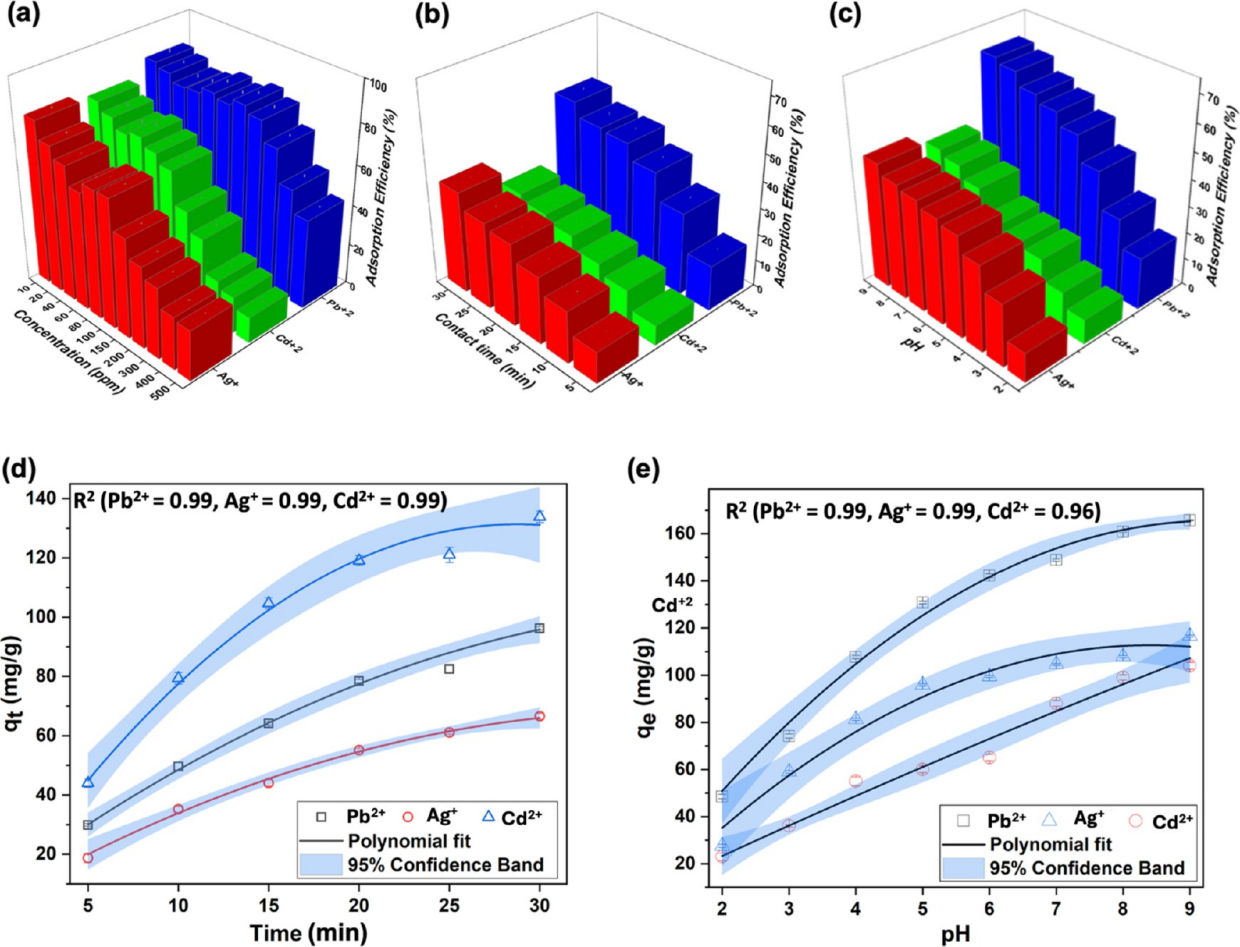
Adsorption efficiency of Fe(III)-TA sorbents for Ag^+^, Cd^2+^, and Pb^2+^ with respect to (a) initial concentration of heavy-metal ions ranged from 10 to 500 ppm, (b) adsorbent–adsorbate contact time for the heavy-metal-ion concentration at 500 ppm, and (c) initial pH of heavy-metal-ion solutions with concentration of 500 ppm. Comparison plots of average adsorption capacities of Fe(III)-TA for Ag^+^, Cd^2+^, and Pb^2+^ with respect to (d) adsorbent–adsorbate contact time and (e) initial pH of the heavy-metal-ion solutions at a concentration of 500 ppm.

Employing a fixed-bed adsorption method, the adsorption equilibrium studies of the sorbent for three heavy-metal-ion contaminants, Pb^2+^, Cd^2+^, and Ag^+^, were conducted using synthetic water samples with initial heavy-metal-ion concentration, ranging from 10 to 500 ppm. Maintaining the adsorbent–adsorbate contact time at 30 min, the percent adsorption efficiencies of heavy-metal ions were obtained from their respective adsorption equilibrium data and are shown in Figure 1a. With the increase of each heavy-metal-ion concentration, we observed a gradual decrease in the adsorption efficiency, evidencing concentration-dependent adsorption of heavy-metal ions onto the sorbent. At the initial heavy-metal-ion concentration of 10 ppm, the sorbent exhibits average adsorption efficiencies of 81, 77, and 85% for Ag^+^, Cd^2+^, and Pb^2+^, respectively. However, at the heavy-metal-ion concentration of 500 ppm, average adsorption efficiencies exhibit a significant reduction to 25, 12, and 44% for Ag^+^, Cd^2+^, and Pb^2+^, respectively. In comparison to the adsorption efficiencies of the sorbent for Ag^+^ and Cd^2+^, the sorbent shows a higher affinity for Pb^2+^ ions. Nonetheless, the gradual decrease in adsorption efficiencies observed with respect to the gradual increase in adsorbate concentration relates to the batch adsorption process, where mass transfer forces between adsorbate and adsorbent’s active sites contribute to the adsorption process.^33−36^ At the low heavy-metal-ion concentration, the availability of vacant pores and binding sites on the sorbent is high, resulting in higher fractional adsorption and mass transfer of heavy-metal ions. With the increase of heavy-metal-ion concentration, available binding sites of the sorbent decrease, resulting in less mass transfer forces between adsorbates and the adsorbent’s active sites. At 500 ppm, eventually, sorbent’s active sites become saturated, hindering the mass transfer of heavy-metal ions, leading to low adsorption efficiencies.

The contact time-dependent adsorption efficiencies obtained for all three heavy-metal-ion solutions with a concentration of 500 ppm (Figure 1b) exhibit a slight increase in adsorption efficiencies, reaching the maximum average adsorption efficiencies of 38, 26, and 52% for Ag^+^, Cd^2+^, and Pb^2+^, respectively, over 30 min of contact time. The results convey that the active sites of the sorbent become saturated with adsorbates within a 30 min contact time, reaching its maximum adsorption efficiencies for respective heavy metals. The pH-dependent studies of the sorbent conducted for the adsorption of heavy-metal ions, with a concentration of 500 ppm, reveal the effect of the amphoteric nature of Fe(III)-TA, confirming that the adsorption of heavy-metal ions depends on the pH of the water samples. As depicted in Figure 1c, the adsorption efficiencies of the adsorbent for all three heavy metals show a gradual increase with the change in pH from acidic to basic, yielding average adsorption efficiency of 38, 44, and 66% for Ag^+^, Cd^2+^, and Pb^2+^, at pH 9, respectively. At basic pH conditions, hydroxyl groups in peripheral catechol units of the sorbent deprotonate, yielding a highly negatively charged sorbent surface. Thus, the adsorption efficiency of the sorbents for heavy-metal ions significantly increases due to the electrostatic attractive forces between the sorbent’s surface and positively charged heavy-metal ions.

Moreover, studies on the time-dependent adsorption and the effect of initial pH of heavy-metal-ion solutions reveal the sorbent’s maximum equilibrium adsorption capacities for three heavy-metal ions (Figure 1d,e). As illustrated in Figure 1d, the adsorption capacity of the sorbent increases with the contact time until the sorbent’s surface becomes saturated with the contaminants. At the initial stage, the rate of heavy-metal-ion adsorption is high, where more ions are adsorbed onto the readily available active sites. As time progresses, the adsorption rate slows and reaches its equilibrium, occupying all available active sites of the sorbent. Over the period of 30 min, the maximum adsorption capacity (*q*_m_) of the sorbent for Ag^+^, Cd^2+^, and Pb^2+^ reaches 96.25, 66.54, and 133.83 mg/g, with the regression coefficient, *R*^2^ = 0.99, respectively. Thus, a time-dependent adsorption study reveals the nature of the adsorption process of Fe(III)-TA sorbents, confirming the optimal contact time for the maximum adsorption capacities for three heavy-metal ions.

The effect of initial pH on the sorbent’s maximum adsorption capacities (*q*_m_) for three heavy-metal ions increases with the increasing initial heavy-metal-ion solution pH from 2 to 9. As shown in Figure 1e, With the increase of pH from 2 to 9, the adsorption capacities significantly increased from 27.56 to 116.57 mg/g for Ag^+^, 23.06 to 104.04 mg/g for Cd^2+^, and 48.47 to 165.66 mg/g for Pb^2+^, respectively. It is also worth noting that at neutral pH (pH = 7) of heavy-metal solutions, the sorbents show a significant heavy metals removal efficiency, with a *q*_e_ of 104.55, 88.04, and 148.85 mg/g for Ag^+^, Cd^2+^, and Pb^2+^, respectively. As described before, the significant increase in *q*_m_ for each heavy metal with respect to their initial solution pH is expected due to the deprotonation of residual hydroxyl groups in catechol units, yielding a highly negative charge surface thereby enhancing electrostatic interactions between the sorbent surface and heavy-metal ions. The pH studies further support the sorbent’s suitability for remediating different types of water resources with pH ranging from 2 to 9.0.

### 2.2 Adsorption Equilibrium Isotherm Models

The mechanistic insight into the sorbent’s adsorption of heavy-metal ions in synthetic water solutions was investigated using well-known adsorption isotherm models; Langmuir, Freundlich, and Temkin models.^37−39^ The nonlinear and linear forms of the Langmuir isotherm model, depicted by eqs 1 and 2, describe the monolayer adsorption process,^37^ in which the magnitude of Langmuir equilibrium constant, *K*_L_ conveys the adsorption energy of the sorbent’s active sites,^37^ revealing the affinity for each heavy-metal ions.
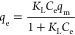
1
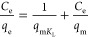
2where *q*_e_ (mg/g) is the equilibrium adsorption capacity; *q*_m_ (mg/g) is the maximum adsorption capacity; *C*_e_ (mg/L) is the concentration of lithium ions when the adsorption reaches equilibrium; and *K*_L_ (L/mg) is the Langmuir equilibrium constant.

The Freundlich adsorption isotherm model, expressed by eqs 3 and 4, typically describes the adsorption of the analyte by a sorbent with the heterogeneous surface, having active sites of variable binding affinities for analytes.^38^ Thus, the adsorption of analytes occurs beyond monolayer adsorption and promotes heterogeneous adsorption.
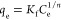
3
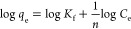
4where *K*_f_ is the Freundlich isotherm constant [mg/g(L/mg)^1/*n*^] and *n* is the adsorption intensity. The magnitude of *K*_f_ reflects the multilayer adsorption capacity and the adsorption intensity, *n*, conveys the heterogeneity of the sorbent.^40^

The Temkin adsorption isotherm model provides greater insight into the interaction between adsorbent and adsorbate, but the validity of the model depends on the intermediate range of analytes concentration.^39^ The liner form of the isotherm is expressed by eq 5.^39^
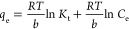
5where *K*_t_ is the equilibrium binding constant (L/g), which represents the maximum binding energy, *b* is the heat of adsorption (J/mol), *T* is the temperature in Kelvin, and *R* is the universal gas constant (8.314 J/K.mol). The plot of *q*_e_ vs ln *C*_e_ produces a slope and intercept for the derivation of *b* and *K*_t_, respectively.

**Figure 2:**
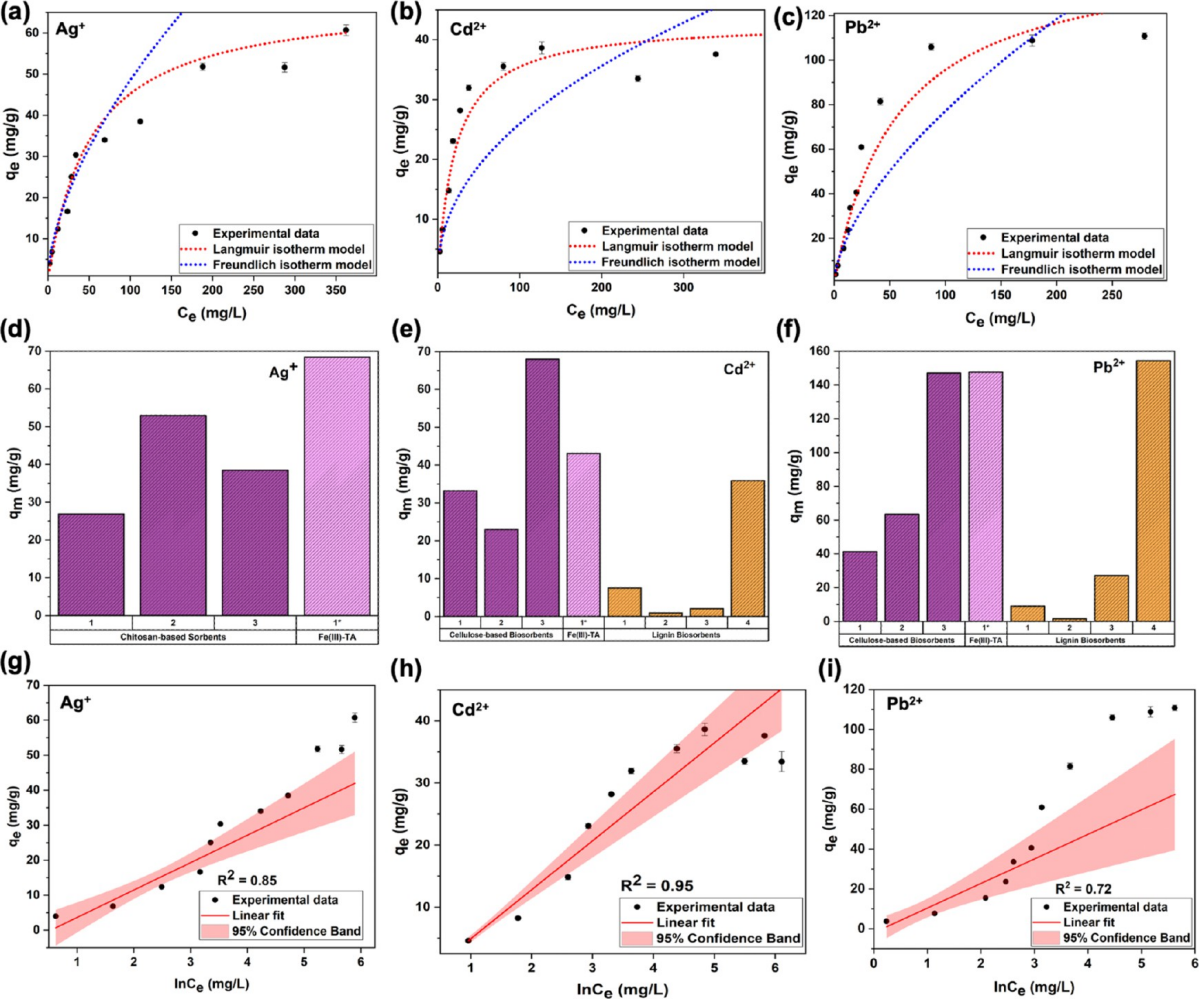
Langmuir and Freundlich adsorption isotherm plots for (a) Ag^+^, (b) Cd^2+^, (c) Pb^2+^. Comparison of maximum adsorption capacities (*q*_m_) of Fe(III)-TA with current biobased sorbents for (d) Ag^+^, (e) Cd^2+^, (f) Pb^2+^. Temkin adsorption isotherm plots for (g) Ag^+^, (h) Cd^2+^, and (i) Pb^2+^.

The adsorption isotherm plots obtained by fitting the adsorption equilibrium data to Langmuir, Freundlich, and Temkin for Ag^+^, Cd^2+^, and Pb^2+^ heavy-metal ions are depicted in Figure 2, and their respective adsorption isotherm parameters are summarized in Table 1. The adsorption isotherm data collected for all three heavy-metal ions in the concentration range of 10–500 ppm fits well with the Langmuir model (Figure 2a–c), shown by higher regression coefficients (*R*^2^ > 0.96), assuming a monolayer coverage and no interactions of adsorbates with neighboring sites. The maximum Langmuir adsorption capacities (*q*_m_) for Ag^+^, Cd^2+^, and Pb^2+^ were calculated to be 68.41, 43.08, and 147.66 mg/g. Agreeing with the experimental maximum adsorption capacity for Pb^2+^ ions, its Langmuir maximum adsorption capacity is significantly higher compared to the Langmuir maximum adsorption capacities for Ag^+^ and Cd^2+^, confirming the sorbent’s higher affinity for Pb^2+^ ions over other two heavy-metal ions. Comparatively, the Langmuir maximum adsorption capacity of Fe(III)-TA sorbent for Ag^+^ is higher than most pertinent literature on chitosan-based adsorbents^41,42^ (Figure 2d and Table S1). Moreover, a comparative collection provided in Figure 2e,f and Table S1, confirms the maximum adsorption capacities for Cd^2+^ and Pb^2+^ are higher than most of the previously reported lignin-based sorbents^43−48^ and cellulose-based sorbents.^49,50^ The Langmuir equilibrium constants (*K*_L_) for Ag^+^ and Pb^2+^ were found to be 0.019 L/g, implying that the monolayer adsorption equilibrium of both metal cations most likely reaches the same time, representing similar adsorption energy for the adsorption of both metal ions onto active sorption sites. The *K*_L_ for Cd^2+^ was calculated to be 0.046 L/g and is comparatively higher, evidencing somewhat higher adsorption energy for the monolayer adsorption of Cd^2+^ ions onto the sorbent’s active sites.

**Table 1:** Adsorption Isotherm Parameters for the Adsorption of Ag^+^, Cd^2+^, and Pb^2+^ onto Fe(III)-TA Sorbents^a^

		adsorbate		
model	parameters	Ag^+^	Cd^2+^	Pb^2+^
Langmuir isotherm	*K*_L_ (L/g)	0.019	0.046	0.019
	*q*_m(cal)_ (mg/g)	68.41	43.08	147.66
	*q*_m(exp)_ (mg/g)	60.70	38.62	110.84
	*R*^2^	0.98	0.97	0.96
Freundlich isotherm	*K*_f_	3.101/1.928^a^	3.160/2.206^a^	4.706/2.773^†^
	*n*	1.67/1.31	2.18/1.30^a^	1.63/1.10^†^
	*R*^2^	0.93	0.76/0.99^a^	0.84/0.97^†^
Temkin isotherm	*K*_t_ (L/g)	0.586/0.649*	0.685/0.696*	0.854/0.848*
	*b* (kJ/mol)	0.316/19.20*	0.312/12.13*	0.200/22.28*
	*R*^2^	0.85	0.95	0.72

a ^†^For low adsorbates concentration range (0–50 ppm concentration of Ag^+^, Cd^2+^, and Pb^2+^); *Revised Temkin parameters calculated from Temkin eq 7 and for *q*_m_, experimental maximum adsorption capacities were used.

Among the plots of adsorption isotherm data fitted to Freundlich models for three heavy-metal ions, the model agrees with the adsorption of Ag^+^ ions with a high *R*^2^ (0.93), evidencing multilayer adsorption of Ag^+^ ions onto the sorbent surface over low-to-intermediate Ag^+^ ions concentration range. However, adsorption isotherms of the other two heavy-metal ions fitted to the model resulted in low *R*^2^ values (*R*^2^ = 0.76 and 0.84 for Cd^2+^ and Pb^2+^, respectively) for the low-to-intermediate adsorbate concentration range. A relatively low *K*_f_ and *n* values for Ag^+^ also support the multilayer adsorption, favoring the low to intermediate range of Ag^+^ ions concentration (10 to 150 ppm), whereas Cd^2+^ and Pb^2+^ adsorptions are unfavorable over the concentration range, yielding rather higher *K*_f_ for Cd^2+^ and Pb^2+^ (Table 1). To gain more insight into the multilayer adsorption for three heavy-metal ions at a low adsorbate concentration range (<50 ppm), the Freundlich isotherm plots were obtained and are depicted in Figure S1. The respective isotherm parameters are summarized in Table 1. The adsorption isotherm data collected for all three heavy-metal ions over the low absorbate concentration range agree with the Freundlich model (Figure S1). More interestingly, Cd^2+^ and Pb^2+^ ions favor the multilayer adsorption at lower concentrations, with high *R*^2^ (Table 1, *R*^2*^). The considerably low *K*_f_^*^ and n* values at the low adsorbate concentration range further support the multilayer adsorption of Ag^+^, Cd^2+^, and Pb^2+^ ions onto the sorbent’s active sites (Table 1).

The linear form of Temkin isotherm plots, depicted in Figure 2g–i for all three heavy-metal ions over the concentration range of 10–500 ppm, reveal that the adsorption isotherms of Ag^+^ and Pb^2+^ ions display a somewhat poor fit for the linear form of the model with very low *R*^2^, but, comparatively, Cd^2+^ adsorption isotherm data obeys the linear form of the model, with *R*^2^ at 95% confidence level. As summarized in Table 1, the Temkin equilibrium binding constant (*K*_t_) calculated for each heavy-metal-ion adsorption is <1 and their respective heat of adsorption energies (*b*) is calculated to be 316.2, 312.9, and 200.8 J/mol, for Ag^+^, Cd^2+^, and Pb^2+^, respectively. The adsorption energies for Ag^+^ and Cd^2+^ closely lie in the same range. However, the adsorption energy for Pb^2+^ is the lowest but theoretically should be higher compared to the adsorption energies for Ag^+^ and Cd^2+^ as Pb^2+^ ions exhibit the highest adsorption onto the sorbent surface, yielding the highest maximum adsorption capacity. Furthermore, at ambient temperature, the adsorption energies for all three heavy-metal ions are <1 kJ/mol, implying poor physisorption of analytes onto the sorbent’s active sites, due to the poorly obeying linear form of the Temkin model (eq 5). Thus, we cannot use the linear form of the Temkin model to accurately describe the adsorption isotherm profiles for the three heavy-metal ions.

As stated in prior literature,^51^ the linear form of the Temkin equation (eq 5), displays two critical issues: dimensionally inconsistent formulation, and the range of validity. Equation 5 is typically valid for intermediate adsorption capacity values; thus, it poorly describes the adsorption process for the entire range of an observed isotherm profile.^51^ Overcoming the dimensional inconsistency of the Temkin eq 5, it was revised by taking fractional coverage (*q*_e_/*q*_m_) into the account instead of just *q*_e_ (eq 6).^51^
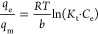
6

The revised Temkin eq 6 corrects the left-hand side of eq 5, which has units of mg/g, and the right-hand side of eq 5, which is unitless. The logarithmic term must be dimensionless, and the prelogarithmic factor *RT*/*b* is also a dimensionless number (*R*, *T*, and *b* in units of J mol^–1^ K^–1^, K, and J mol^–1^, respectively). The dimensional inconsistency of the Temkin eq 5 is now being corrected, and the revised *K*_t_^†^ and *b*^†^ are summarized in Table 1.

**Figure 3:**
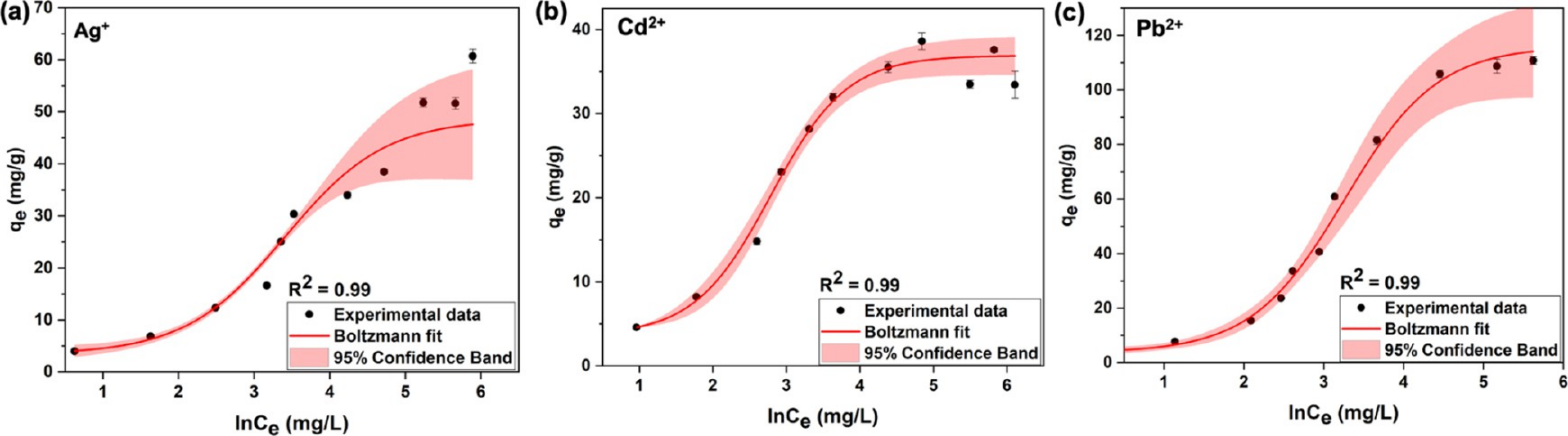
Sigmoidal adsorption isotherm (S_II_-type) plots for (a) Ag^+^, (b) Cd^2+^, and (c) Pb^2+^.

With the correction of dimensional inconsistency, the heat of adsorption for analytes increases from Cd^2+^ < Ag^+^ < Pb^2+^, where adsorbate Pb^2+^ exhibits the highest heat of adsorption, agreeing with its highest experimental adsorption capacity compared to the other two analytes. Furthermore, it is well known that the heat of adsorption lower than 80 kJ/mol normally favors the physisorption process, and for chemisorption, it is higher than this limit.^52,53^ Thus, the theoretical values calculated from eq 6 for their respective heat of adsorption energies reflect that each analyte could adsorb onto the sorbent surface via physisorption at ambient conditions. However, considering the full range of validity of the Temkin model, our experimental adsorption data collected for each analyte concentration poorly fits both forms of the Temkin model. Thus, the findings of our studies cannot be generalized, and the experimental adsorption isotherm data suggest that the data displays a different adsorption isotherm behavior. Therefore, addressing the shortcoming of the Temkin model’s range of analytes concentration validity, we derived an adsorption isotherm model that fits the full range of analytes concentrations. The adsorption isotherm profiles (Figure 3) of Ag^+^, Cd^2+^, and Pb^2+^ ions onto the sorbent follow the sigmoidal Boltzmann function, shown in eq 7, with *R*^2^ > 0.95, suggesting a sigmoidal type (S-type) adsorption pathway, like that observed for some other solid-phase multilayer adsorption processes.^54^ All three adsorption isotherm profiles describe the S_II_-type isotherms, which follow positive adsorption tendency, indicating either a dependence of surface adsorption on the bulk composition or the existence of a micropore-filling adsorption mechanism.^54^ Thus, we can derive a new sigmoidal adsorption isotherm model, that describes the adsorption process for a S_II_-type adsorption isotherm, with the validity for a full range of analyte concentration, by converging eq 7 to eq 8.
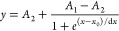
7where *A*_1_ and *A*_2_ are the final and initial values of the sigmoidal parameters, respectively, which represent the maximum adsorption capacity (*q*_m_) and initial adsorption capacity (*q*_0_) of the adsorption isotherm profiles. The center of the linear phase, *x*_0_, which can be denoted as the intermediate concentration, *C*_0_, (*x*) is the final value at the steady phase of the isotherm and can be substituted by the highest analyte concentration, *C*_e_, and *dx* is the concentration-dependent constant, describing the equilibrium binding constant, *K*_t_. Thus, the new adsorption isotherm model, describing the full range of analyte concentration, can be represented by eq 8. Considering the dimensional consistency, eq 8 can be further refined by adding the dimensional constant, *f*, that describes the heat of adsorption, *b*, for analytes at a specific temperature, *T* (in Kelvin), and the gas constant, *R*. Thus, eq 8 can be written as eq 9, where 1/*f* = *RT*/*b*.
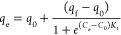
8
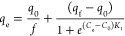
9

The binding constants (*K*_t_) calculated from the S_II_-type adsorption isotherm plots are to be 0.664, 0.518, and 0.583 L/g for Ag^+^, Cd^2+^, and Pb^2+^, respectively, and are comparatively lower than the binding constants obtained from original and revised Temkin models (eqs 5 and 6). The lower binding constants further convey the excellent conformity of the sigmoidal adsorption isotherm model, favoring the analytes’ dependence on surface adsorption at room temperature followed by micropore filling of analytes. The well-obeyed sigmoidal nature of S_II_-type adsorption isotherms also implies the lateral interactions between the adsorbed species during the pore filling of micropores.^55^ In our case, Fe(III)-TA sorbents are highly porous with the distribution of micropore width, ranging from 2 < 100 nm,^31^ enabling the pore filling of micropores. For example, hydrophobic microporous solids such as aluminum phosphate (ALPO), silicon aluminum phosphate (SAPO), and similar zeolite analogue materials,^56−60^ metal–organic frameworks (MOFs),^61^ and activated carbon^62,63^ obey S-type adsorption isotherms due to the pore filling of micropores.

The heat of adsorption calculated for each adsorbate is in the range of 2–4 kJ/mol and suggests the favorable lateral interactions between adsorbates and adsorbate–adsorbent interactions, leading to micropore filling at room temperature. The heat of adsorption (*b*) values calculated are 2.898, 4.084, and 2.842 kJ/mol for Ag^+^, Cd^2+^, and Pb^2+^, respectively. Furthermore, these moderate heat of adsorption values, compared to the heat of adsorption values calculated from both Temkin models where either heat of adsorption is significantly lower (<0.5 kJ/mol) or significantly higher (>5 kJ/mol), evidence the energetically favorable physisorption and micropore filling at room temperature. However, further insight is necessary for definitive conclusion on the physisorption followed by micropore filling of analytes during the adsorption process. The temperature-dependent adsorption of heavy-metal ions onto the sorbent discussed in a follow-up section on the thermodynamic studies will also allow us to understand the physisorption and micropore filling of analytes.

#### 2.2.1 Adsorption Kinetic Isotherm Models

The kinetic isotherm profiles of heavy-metal ions reveal the adsorption kinetics of the sorbent, revealing the mechanistic pathway for adsorbing heavy-metal ions Ag^+^, Cd^2+^, and Pb^2+^. Kinetic isotherm data collected for all three heavy metals was analyzed using pseudo-first-order, pseudo-second-order, and Elovich kinetic models. A pseudo-first-order model describes simple kinetic adsorption of an analyte from its nonlinear form (eq 10)^64^ and linear form (eq 11).^65^ A pseudo-second-order model represents the adsorption equilibrium capacity and can be represented in eqs 13 and 14.^65,66^
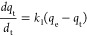
10
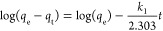
11where *k*_1_ is the rate constant for the pseudo-first-order adsorption (min^–1^), *q*_e_ is the adsorption capacity at equilibrium (mg/g), and *q_t_* is the adsorption capacity at time *t*, (mg/g).
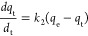
12
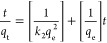
13where *k*_2_ is the rate constant of the pseudo-second-order adsorption.

The Elovich model describes the chemical adsorption in nature, representing sorbents with heterogeneous adsorption surfaces.^67^ Its nonlinear and linear forms can be expressed from eqs 14 and 15.^66^
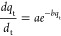
14
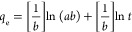
15where *a* and *b* are the initial adsorption rate (mg/(g·min)) and desorption constant (g/mg), respectively.

**Figure 4:**
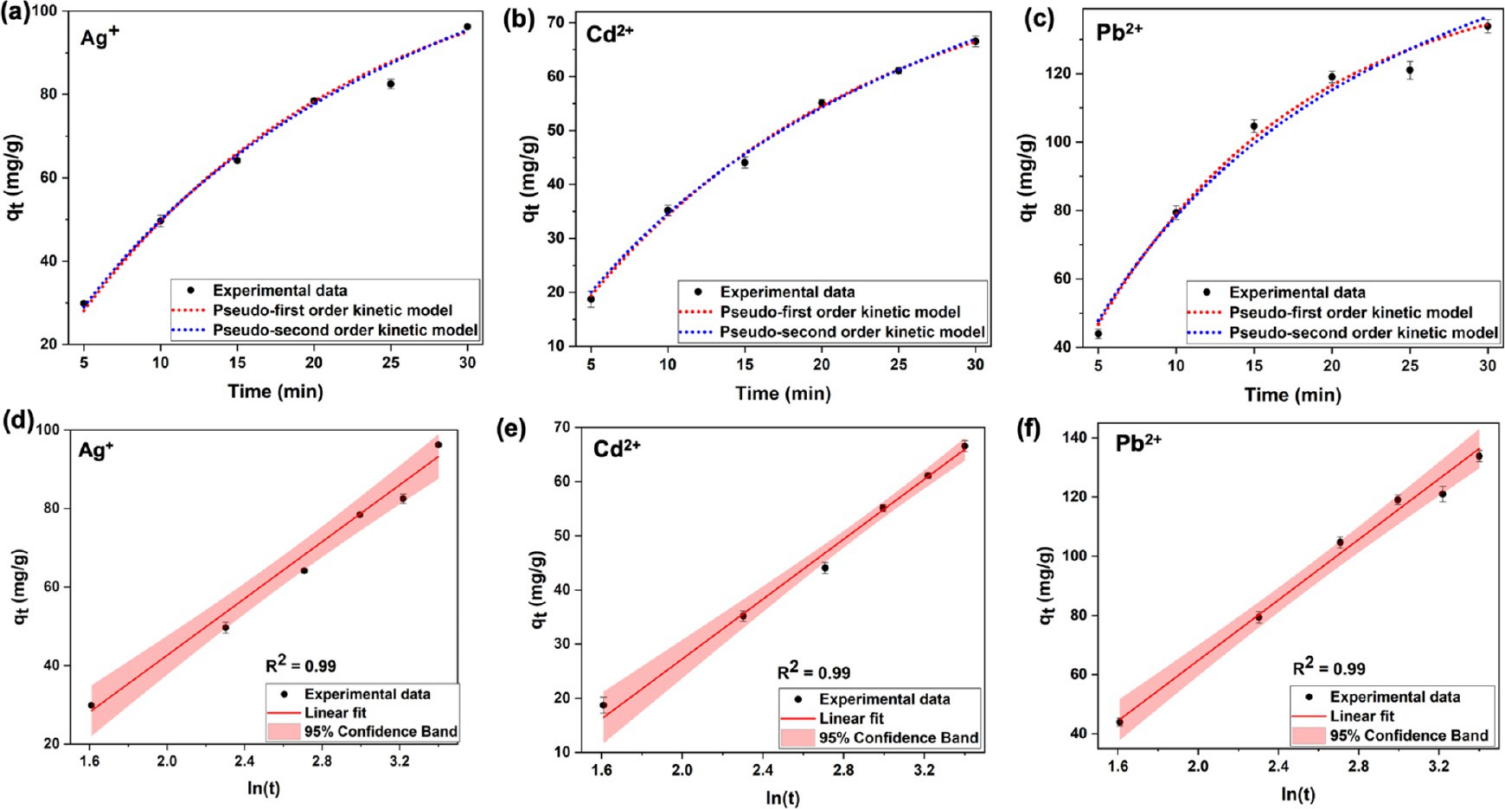
Pseudo-first- and pseudo-second-order nonlinear kinetic isotherm profiles for (a) Ag^+^, (b) Cd^2+^, and (c) Pb^2+^. Elovich kinetic isotherm plots for (d) Ag^+^, (e) Cd^2+^, and (f) Pb^2+^.

The pseudo-first-order and pseudo-second-order kinetic isotherms obtained from the time-dependent adsorption isotherm data of three heavy-metal ions are shown in Figure 4a–c. The adsorption kinetics of the sorbent for three heavy-metal ions obey both models (*R*^2^ > 0.99), implying that the kinetic adsorption process of three analytes occurs via physisorption and chemisorption, respectively. The adsorption kinetic parameters calculated from both models are summarized in Table 2. The kinetic constants (*k*_1_) for the pseudo-first-order kinetic adsorption model of Ag^+^ and Cd^2+^ are in the same range (*k*_1_ = 0.053–0.054 min^–1^). However, their equilibrium adsorption capacities (*q*_e_ = 118.22 mg/g for Ag^+^ and 83.64 mg/g for Cd^2+^) calculated from their respective nonlinear kinetic isotherm plots (Figure 4) deviate significantly, suggesting the presence of heterogeneous binding sites with selective binding affinity for Ag^+^ over Cd^2+^ ions. The kinetic constant calculated for Pb^2+^ is comparatively higher (*k*_1_ = 0.074 min^–1^), evidencing faster adsorption kinetic of Pb^2+^ onto the sorbent’s active sites, resulting in higher equilibrium capacity, *q*_e_ = 150.64 mg/g.

**Table 2:** Kinetic Adsorption Isotherm Parameters Obtained for Pseudo-First-Order, Pseudo-Second-Order, and Elovich Models

	Pseudo-first order			Pseudo-second order		
adsorbate	*k*_1_ (min^–1^)	*q*_e,calc_ (mg/g)	*R*^2^	*k*_2_ (g/mg·min) × 10^–4^ ± 10^–5^	*q*_e,calc_ (mg/g)	*R*^2^
Ag^+^	0.054 ± 0.005	118.2 ± 6.055	0.99	2.242 ± 3.459	176.1 ± 9.600	0.99
Cd^2+^	0.053 ± 0.004	83.64 ± 3.236	0.99	3.014 ± 5.088	125.8 ± 7.470	0.99
Pb^2+^	0.074 ± 0.007	150.6 ± 6.500	0.99	2.597 ± 6.783	217.3 ± 18.85	0.98

Elovich Model			
adsorbate	Ag^+^	Cd^2+^	Pb^2+^
*a* (mg/g·min)	0.012	0.013	0.009
*b* (g/mg*)*	0.027	0.036	0.019

The pseudo-second-order kinetic constants (*k*_2_) obtained for all three heavy-metal ions are in 1/10,000th order, indicating slow chemisorption of heavy-metal ions. It is worth noting that the equilibrium adsorption capacities obtained from the nonlinear form of the pseudo-second-order kinetic plots exhibit considerably higher capacities compared to the adsorption capacities obtained from the nonlinear plots of pseudo-first-order kinetic model. Thus, the results confirm that chemisorption occurs at the sorbent surface, leading to higher adsorption of heavy-metal ions. The kinetic data fitted into the linear form of the Elovich model also confirms the chemisorption of heavy-metal ions, agreeing with the pseudo-second-order kinetic models for chemisorption onto the sorbent’s heterogeneous surface (Figure 4d,e). Table 2 summarizes the Elovich parameters of the adsorption rate constant (*a*) and the desorption constant (*b*). The adsorption rate constants are larger than the pseudo-second-order rate constants, supporting a faster rate of chemisorption via a heterogeneous adsorbent surface. The desorption constants (*b*) are comparable to most natural sorbents for cations adsorption, which follow the Elovich model.^67^

#### 2.2.2 Diffusion-Controlled Kinetic Models

The effect of diffusion and surface reaction mechanisms conjointly describes the kinetics of adsorption. The diffusion models are applicable when the rate-determining step is the mass transfer of adsorbate to the solid surface sites, whereas the pseudo-first-order and pseudo-second-order kinetic models are used for the description of adsorption kinetics when the overall sorption rate is controlled by the rate of surface adsorption, i.e., physisorption and chemisorption. In our case, the adsorption equilibrium studies evidence that the sorbet displays S_II_-type adsorption isotherm and the kinetic adsorption data agrees with the pseudo-first- and pseudo-second-order as well as Elovich models, supporting the involvement of external and internal diffusions of heavy-metal ions, filling the pores of the sorbents. Thus, providing further insight, adsorption kinetic isotherm data was fitted to two diffusion models: an external diffusion model (eq 16)^68^ and an internal diffusion model, also known as the Weber and Morris models (eq 17).^69^
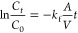
16
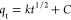
17where *C*_0_, *C*_t_, *A*/*V*, and *t* are the initial analyte concentration, analyte concentration at time *t*, the external sorption area (*A*) to the total solution volume (*V*), and sorption time (*t*), respectively. The external diffusion coefficient *k*_f_ (dm^3^/g·min) can be calculated from the slope of the straight line obtained from eq 16. In eq 17, *k* is the internal diffusion coefficient (mg/g·min^1/2^), which is given from the slope of the linear curve, and *C* is the intercept that represents the boundary layer effect.^70,71^

**Figure 5:**
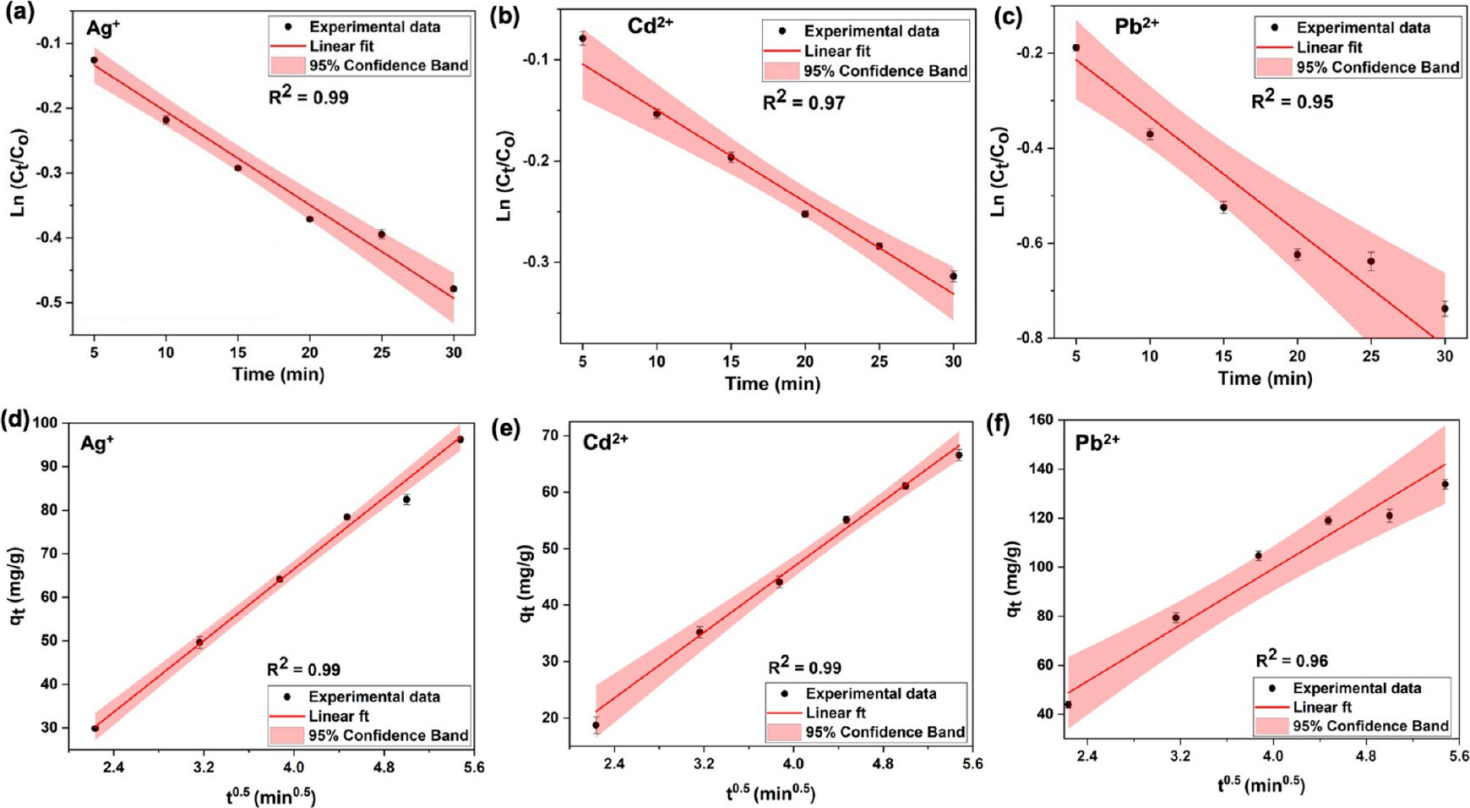
Diffusion-controlled kinetic models: (a–c) External diffusion plots and (d–f) Internal diffusion plots for Ag^+^, Cd^2+^, and Pb^2+^.

As shown in Figure 5a–c, external diffusion plots for three heavy-metal ions are linear throughout the adsorption time, implying that the adsorption of heavy-metal ions is initially driven by surface adsorption followed by external diffusion. The external diffusion coefficients (*k*_f_) were calculated to be 0.007 dm^3^/(g·min) for Ag^+^, 0.004 dm^3^/(g·min) for Cd^2+^, and 0.012 dm^3^/(g·min) for Pb^2+^. Compared to the adsorption kinetic rate constants (*k*_1_ and *k*_2_ in Table 2), the *k*_f_ for each heavy metal lies in between *k*_1_ and *k*_2_ where *k*_1_ > *k*_f_ > *k*_2_, suggesting heavy metals adsorb onto the sorbent’s surface, first via physisorption followed by external mass transfer diffusion, and then the chemisorption. The kinetic adsorption data fitted to the internal diffusion kinetic model based on the Weber–Morris equation (eq 18) exhibit linear profiles for three heavy-metal ions (Figure 5d–f), with >96% confidence (*R*^2^ = 0.99 for Ag^+^ and Cd^2+^ and 0.96 for Pb^2+^).

The internal diffusion coefficients (*k*) for Ag^+^, Cd^2+^, and Pb^2+^ are found to be 20.5, 14.5, and 28.7 mg/(g·min^1/2^), respectively. Having larger *k* values indicates that the intraparticle diffusions of heavy-metal ions are significantly favorable compared to both physisorption and chemisorption. The results convey that the sorbents participate simultaneously in surface diffusion and intraparticle diffusion, evidencing that the mass transfer of heavy-metal ions occurs by diffusion into both the adsorbed solid phase and adsorbent’s pores after the surface adsorption, following physical and chemical mechanisms. Overall, kinetic adsorption isotherm results and the mass transfer diffusion models evidence that the adsorption of three heavy-metal ions occurs via surface adsorption and external and internal mass transfer diffusion, saturating the sorbent’s surface-active sites and its pores.

To understand the absorption efficiency of the sorbents at a very low level of heavy-metal-ion concentrations, we have conducted batch adsorption studies for aqueous solutions with heavy-metal-ion concentrations of 100 and 1000 ppb, and the results are summarized in Table S2. At both heavy-metal-ion concentrations, Fe(III)-TA sorbent exhibits >90% adsorption efficiency for all three heavy metals, compared to lower adsorption efficiencies observed at higher heavy-metal-ion concentrations (10–500 ppm). This difference is attributed to the excess active sites for analytes, facilitating effective adsorption with higher efficiency at lower concentrations of analytes. The sorbents adsorb Ag^+^ and Pb^2+^ with 99% adsorption efficiency for water samples with 100 ppb metal ion concentration, demonstrating the applicability of our sorbents for the removal of heavy metals from a variety of surface water sources to meet drinking water standards.

#### 2.2.3 Thermodynamic Studies of Fe(III)-TA for Heavy-Metal-Ion Adsorption

To understand the effect of temperature on the adsorption efficiency at ambient temperature (25 °C) versus at higher temperatures, we have conducted batch adsorption studies of the sorbent at four different temperatures, starting from 25 °C (298 K), with 10 °C incremental up to 55 °C (328 K). Taking Pb^2+^ as our model analyte, adsorption equilibrium isotherm data collected at each temperature was analyzed using Van‘t Hoff equation,^72^ which describes the thermodynamic parameters—Gibbs free energy (kJ/mol), enthalpy change (kJ/mol), and entropy change (J/mol K)—during the adsorption equilibrium process. These parameters can be calculated from eqs 18–20.
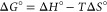
18
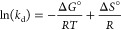
19and
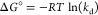
20Where
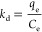
21where *R* is the universal gas constant, 8.314 J K^–1^ mol^–1^, *T* is the temperature in Kelvin, *k*_d_ is the adsorption equilibrium constant (L/g), and *q*_e_ and *C*_e_ are the adsorption capacity (mg/g) and equilibrium concentration (mg/L) in the aqueous phase, respectively. The values of enthalpy (Δ*H*) and entropy (Δ*S*) are derived from the slope and *y*-intercept of the Van’t Hoff plot of ln (*k*_d_) versus 1/*T* presented in eq 19.^73^

**Figure 6:**
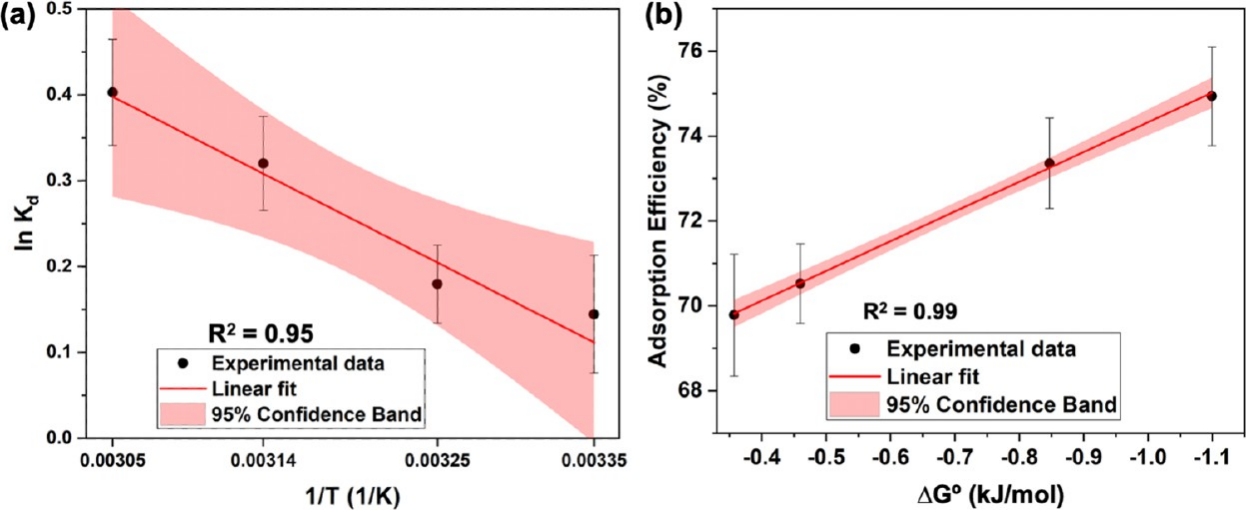
(a) Van’t Hoff plot for the adsorption of Pb^2+^ onto Fe(III)-TA sorbents and (b) adsorption efficiency of Fe(III)-TA sorbents at for Pb^2+^ with respect to Δ*G*°.

The Van’t Hoff plot, and the adsorption efficiency vs Gibbs free energy plot, obtained from the temperature-dependent Pb^2+^ adsorption equilibrium isotherm data are shown in Figure 6a,b, respectively. Table 3 summarizes the temperature-dependent adsorption efficiencies along with the thermodynamic parameters. Although the adsorption efficiencies do not increase significantly with temperature, the linear trend of the Van’t Hoff plot (Figure 6a) indicates that the adsorption of heavy-metal ions onto the sorbent is temperature-dependent. This observation is further supported by the negative values of Δ*G*° for Pb^2+^ adsorption across the temperature range (Table 3), reflecting a thermodynamically spontaneous nature of the analyte’s adsorption process. Additionally, the increasing negativity of Δ*G*° with rising temperature correlates with enhanced adsorption efficiency as reflected in Figure 6b, demonstrating the favorable adsorption of analytes at higher temperatures.^74^ Correlating with the S_II_-type absorption isotherm behavior that was observed at room temperature, suggesting physisorption followed by micropore filling, the thermodynamic parameters also support that analyte adsorption occurs via physisorption followed by micropore filling. At higher temperatures, micropore filling is energetically favorable, resulting in a linear increase of the adsorption efficiency. As summarized in Table 3, the positive enthalpy change (Δ*H*) and the positive entropy change (Δ*S*) also reflect the endothermic nature of the adsorption process, which is energetically favorable at higher temperatures, and the analyte’s adsorption becomes rather random due to the pore expansion at higher temperature.

**Table 3:** Thermodynamic Parameters and Adsorption Efficiencies of the Sorbent for Pb^2+^

temperature (°C)	25	35	45	55
Δ*G*° (kJ/mol)	–0.358	–0.460	–0.847	–1.099
adsorption efficiency (%)	69.78 ± 1.43	70.52 ± 0.94	73.36 ± 1.06	74.94 ± 1.17
Δ*H*° (kJ/mol)	7.76			
Δ*S*° (J/mol·K)	26.90			

#### 2.2.4 Mechanistic Understanding of the Heavy-Metal-Ion Adsorption

**Figure 7:**
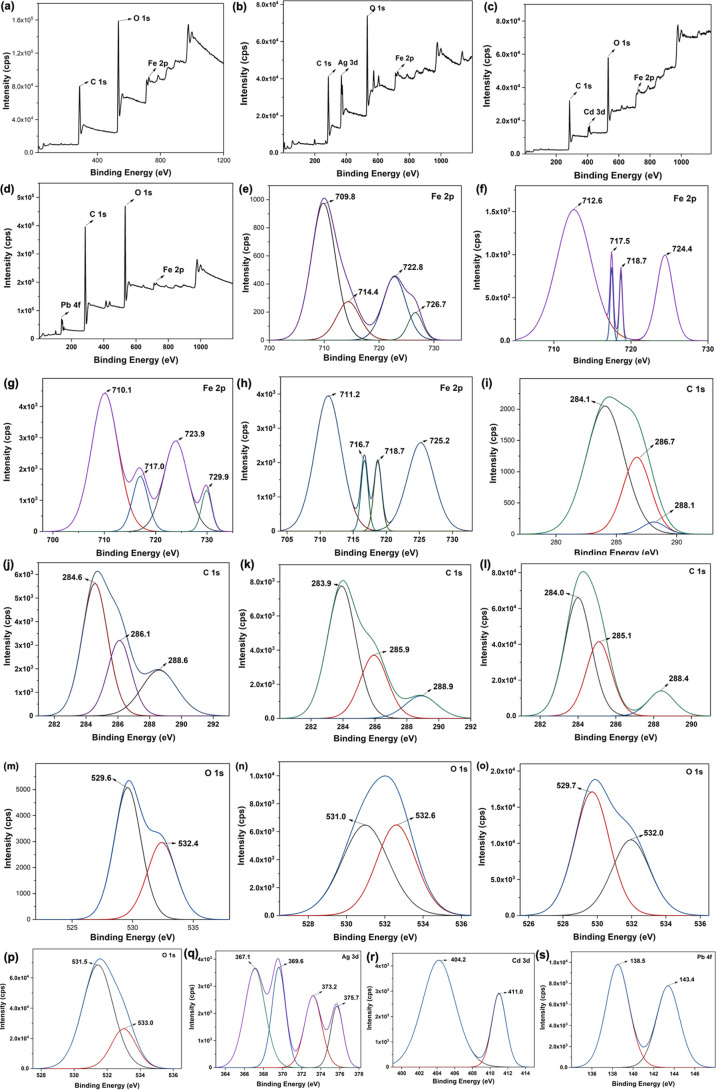
XPS survey spectra of (a) pristine Fe(III)-TA, and used Fe(III)-TA with (b) Ag^+^, (c) Cd^2+^, and (d) Pb^2+^, and their respective binding energy spectra for (e–h) Fe 2p, (i–l) C 1s, (m–p) O 1s, (q) Ag 3d, (r) Cd 3d, and (s) Pb 4f.

To understand the nature of interactions between each adsorbate and the sorbent, we conducted an X-ray photoelectron spectroscopy (XPS) analysis of the sorbent after soaking in each heavy-metal solution with a concentration of 500 ppm, over 30 min. The XPS survey spectra and binding energy spectra of each heavy-metal-ion-adsorbed sorbent are depicted in Figure 7. The retention of the coordination framework of the sorbent confirms the XPS elemental survey spectra of the pristine sorbents and used sorbents with the respective heavy-metal ion (Figure 7a–d). In comparison to the Fe 2p binding energy spectrum of the pristine sorbent, the Fe 2p binding energy spectra of heavy-metal-ion-adsorbed sorbents exhibit noticeable shifts (ca. 2–3 eV) in the binding energies, confirming the chemical environment changes due to the adsorbent’s active sites interactions with each heavy-metal ion. The Fe 2p binding energy spectrum (Figure 7e) of the sorbent exhibits two main peaks at 709.8 and 722.8 eV, and two broader satellite peaks at 714.4 and 726.7 eV, representing Fe^2+^ and Fe^3+^ oxidation states, respectively. In Fe 2p binding energy spectra of Ag^+^ and Pb^2+^ ions adsorbed sorbents (Figure 7f,h); the satellite peaks are well resolved with an additional satellite peak at 718.7 eV but lacking the satellite peak at 726.7 eV. These distinct differences reflect the nature of the chemical environment due to the analyte interactions with the sorbent’s active sites. The Fe 2p binding energy spectrum (Figure 7g) of the Cd^2+^ adsorbed sorbent follows the same peak characteristics as the Fe 2p spectrum of the sorbent, with 2–3 eV shifts in the binding energies of the respective peaks. There are noticeable changes in the chemical environment, reflected from C 1s and O 1s binding energy spectra (Figure 7i–l,m–p) of heavy-metal-ion-adsorbed sorbents, compared to the respective binding energy spectrum of pristine sorbent, confirming the analytes’ interactions with the sorbent. The binding energy spectra of Ag 3d, Cd 3d, and Pb 4f (Figure 7q–s) confirm the adsorption of each analyte onto the sorbent.

**Figure 8:**
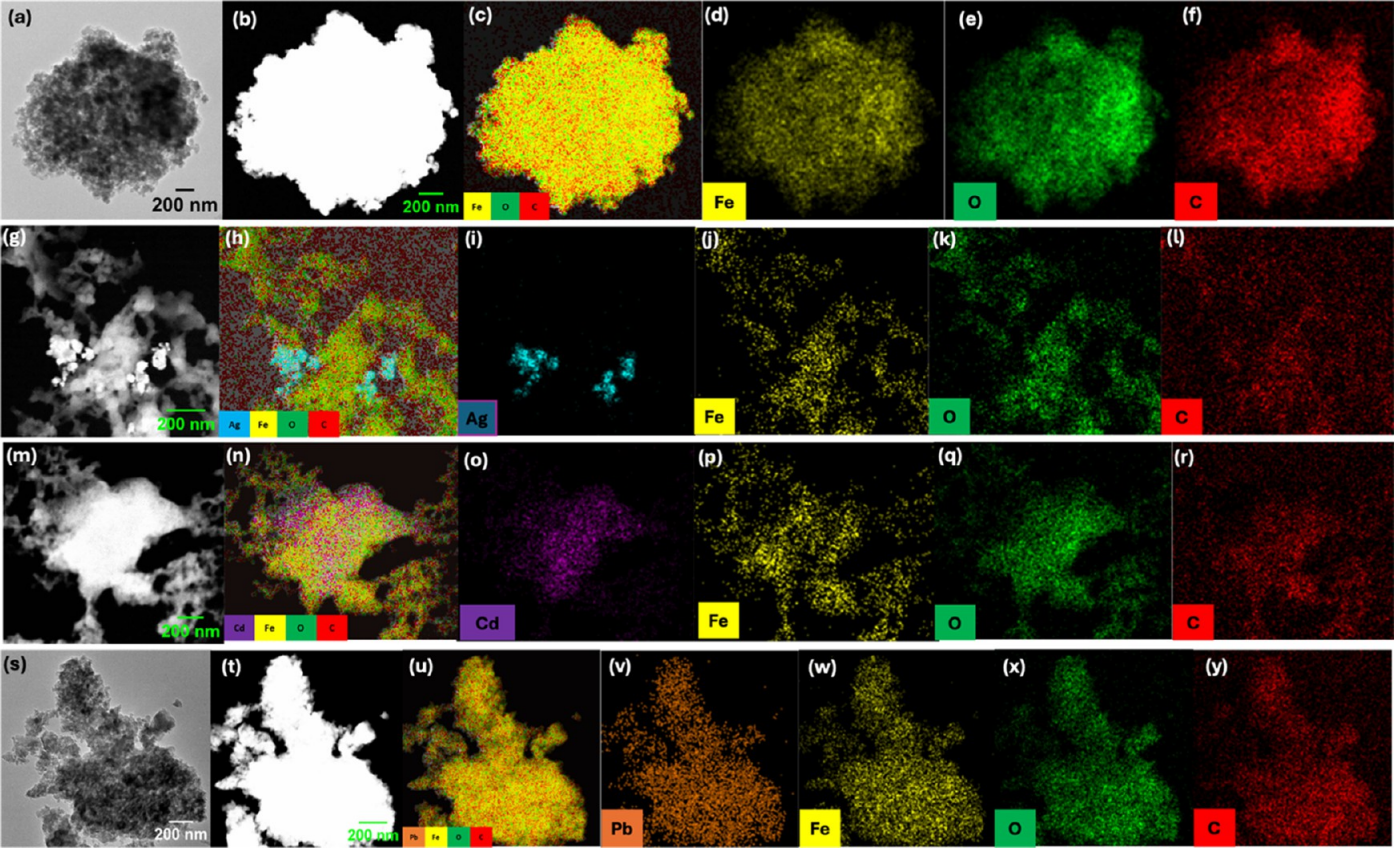
(a) HR-TEM image of Fe-TA taken at 10 kx; (b) ADF-STEM image; (c) EDX overlay elemental map; and (d–f) individual elemental mapping for Fe, O, and C, respectively, for pristine Fe(III)-TA. (g) ADF-STEM image; (h) EDX overlay elemental map; and (i–l) individual elemental mapping for Ag, Fe, O, and C respectively, for Ag^+^ ions adsorbed Fe(III)-TA. (m) ADF-STEM image; (n) EDX overlay elemental map; and (o–r) individual elemental mapping for Cd, Fe, O, and C, respectively, for Cd^2+^ ions adsorbed Fe(III)-TA. (s) HR-TEM image of Pb^2+^ adsorbed Fe(III)-TA taken at 10 kx; (t) ADF-STEM image; (u) EDX overlay elemental map; and (v–y) individual elemental mapping for Pb, Fe, O, and C, respectively, for Pb^2+^ ions adsorbed Fe(III)-TA.

High-resolution transmission electron microscopy (HR-TEM) combined with energy-dispersive X-ray spectroscopy (EDX) analysis reveals the heavy-metal-ion distribution within the sorbents. Heavy-metal-ion-treated sorbents along with the pristine sorbents, visualized from HR-TEM combined with EDX are shown in Figure 8. The TEM image of pristine sorbent, shown in Figure 8a, along with its angular dark-field scanning transmission electron microscopy (ADF-STEM) image (Figure 8b), confirms the porous nature and the corresponding elemental maps (Figure 8c–f) confirm metal oxide nodes (FeO), coordinated tannic acid framework. The ADF-STEM images (Figure 8g,m,t) and the respective elemental distribution maps (Figure 8h–l,n–r,u–y) obtained for Ag^+^, Cd^2+^, and Pb^2+^ ions adsorbed sorbents provide clear evidence to external and internal diffusion of analytes, filling sorbent’s micropores.

**Figure 9:**
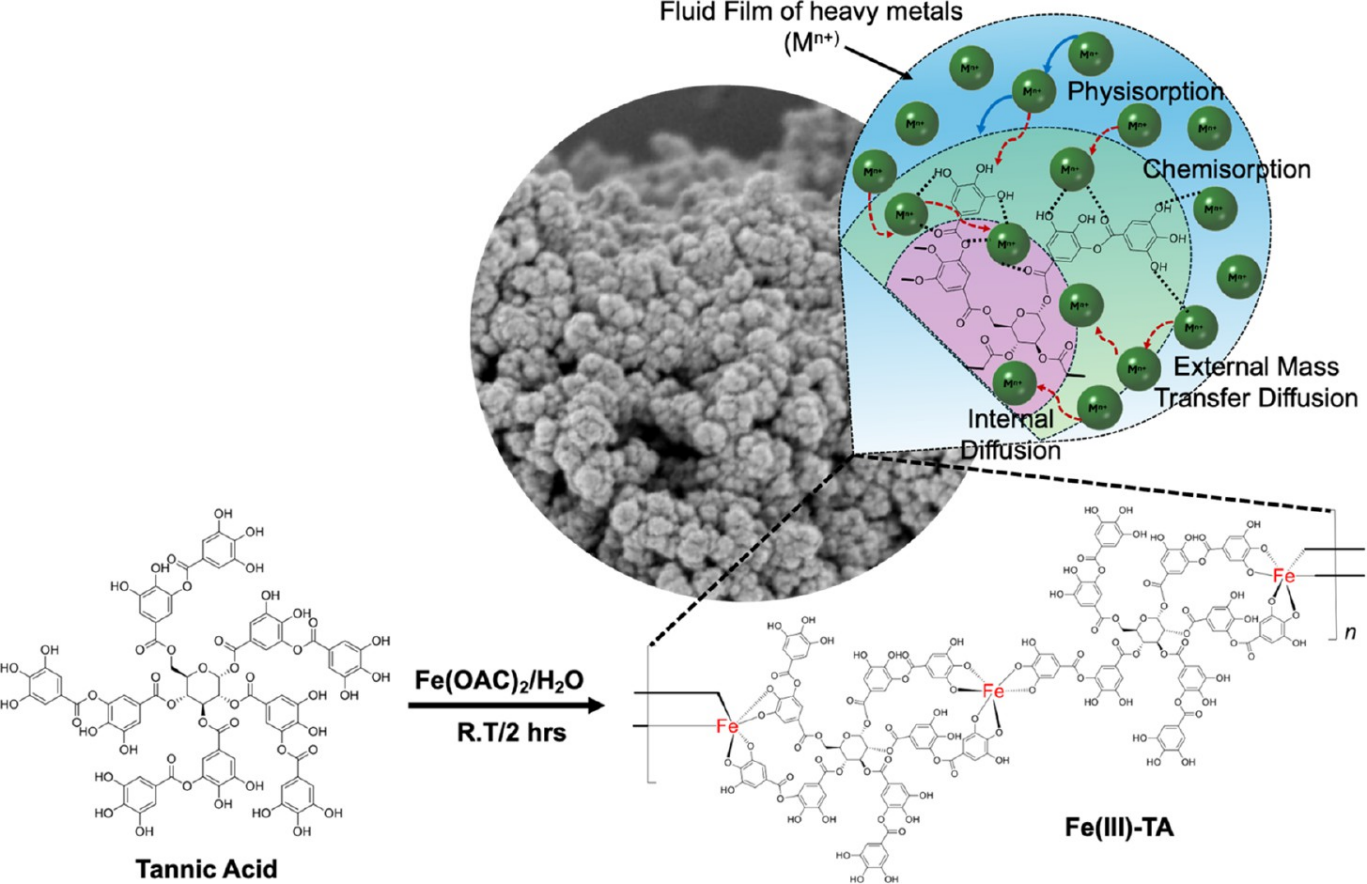
Chemistry of the formation of Fe(III)-TA sorbents and the postulated heavy-metal-ion sieving pathway based on the adsorption isotherm models and diffusion models.

Figure 9 depicts the postulated stepwise heavy-metal-ion adsorption process along with the chemistry of Fe(III)-TA formation. Based on the XPS results combined with HR-TEM images and EDX elemental maps, the heavy-metal-ion adsorption mechanism follows a conjoint surface and diffusion reaction mechanism, which involves physisorption, external mass transfer diffusion, chemisorption, internal mass transfer diffusion and eventually filling the pores. We can postulate that the adsorption mechanism follows first forming a fluid film of analytes on the sorbent’s surface, facilitating the physisorption via electrostatic interactions of heavy-metal ions onto the sorbent’s surface. Following the external mass transfer diffusion process, the chemisorption of heavy-metal ions begins by interacting heavy-metal ions with periphery hydroxyl groups of catechol units and ester groups of pyrogallol units. The nature of the chemical environment changes, reflected from the binding energy spectra of C 1s, O 1s, and Fe 2p support the chemical interactions of metal ions with hydroxyl and ester groups of tannic acid as well as coordinated Fe(III) metal ion nodes. When the surface adsorption reaches its saturation, the internal mass transfer diffusion of heavy-metal ions takes place, filling the pores of the sorbent.

However, it is also possible that heavy-metal-ion adsorption onto the sorbents could follow the ion-exchange mechanism wherein heavy metals slowly can exchange with Fe^2+^/Fe^3+^ cations in the framework. To rule out the involvement of any ion-exchange processes for heavy-metal-ion adsorption, we monitored the concentrations of Fe^2+^, Ag^+^, Cd^2+^, and Pb^2+^ ions in the aqueous phase during the time-dependent adsorption process by correlating the intensity (counts/seconds) of each analyte measured from the ICP analysis to the analyte concentration. The control experiment on pristine sorbents dispersed in DI water exhibits stable Fe^2+^ concentration, accounting for 105–115 count/s due to the trace amount of surface adsorbed metal cations (Figure S2a). In comparison to the control experiment, Fe(III)-TA sorbents soaked in a mixture of heavy-metal-ion solution, over a 30 min period, exhibit a reduction in heavy-metal ions’ intensity while Fe^2+^ intensity remains at a constant level, comparable to the intensity range of the control (Figure S2b,c). Thus, the results confirm that there is no heavy-metal-ion exchange with the framework metal oxide nodes’ iron cations.

### 2.3 Study on Adsorption Desalination

**Figure 10:**
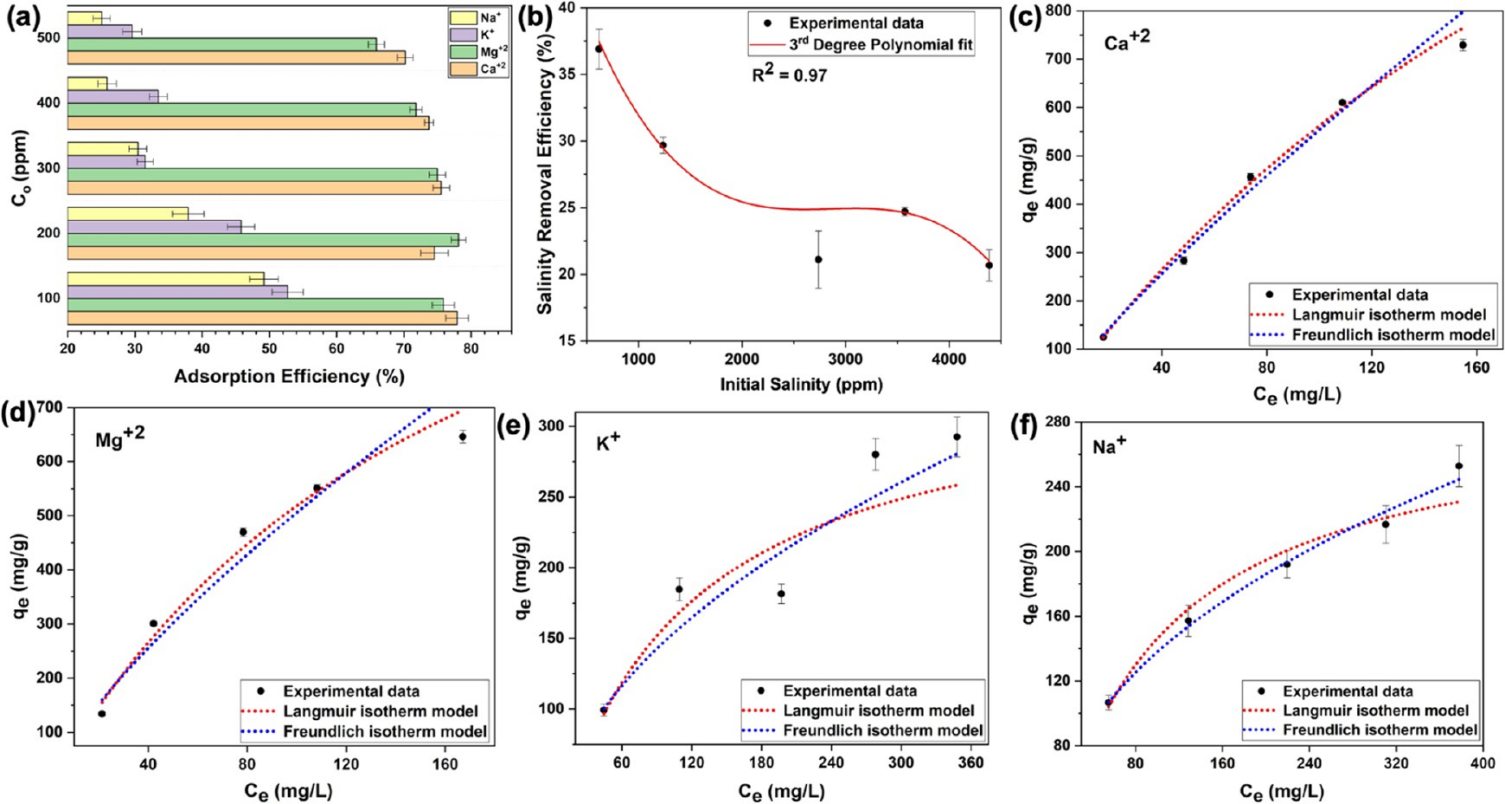
Plots of (a) AD efficiency of alkali and alkaline cations with respect to different brine concentrations (contact time 24 h); (b) salinity removal efficiency of brines; and Langmuir and Freundlich adsorption isotherms for (c) Ca^2+^, (d) Mg^2+^, (e) K^+^, and (f) Na^+^.

Adsorption desalination (AD) studies were conducted using synthetic brine samples and seawater samples, employing a fixed-bed column set up at ambient conditions. The AD efficiency of the pristine sorbents for alkali (Na^+^ and K^+^), and alkaline (Mg^2+^, and Ca^2+^) cations were investigated using synthetic brine samples, with the concentration ranging from 100 to 500 ppm, and the contact time of 24 h. The AD efficiency with respect to the initial concentration of each analyte in brine samples and the total salinity removal efficiency are depicted in Figure 10a,b, respectively. Table 4 summarizes the AD efficiency of each cation along with the salinity removal efficiencies.

**Table 4:** Summary of Adsorption Characteristics, Salinity Removal Efficiency, and Adsorption Isotherms of Pristine Fe(III)-TA Sorbents for Desalination of Brine and Seawater Samples^a^

	Adsorption Dfficiency (%) ± SD				Salinity Removal Efficiency (%)	
brine concentration (ppm)	Ca	Mg	K	Na	initial salinity (ppm)	AD efficiency (%)
100	77.92 ± 1.68	75.86 ± 1.64	52.71 ± 2.30	49.18 ± 2.11	618	36.90 ± 1.49
200	74.55 ± 2.05	78.13 ± 1.12	45.80 ± 2.02	37.93 ± 2.34	1236	29.70 ± 0.64
300	75.56 ± 1.24	74.97 ± 1.24	31.54 ± 1.18	30.45 ± 1.32	2737	21.10 ± 2.15
400	73.72 ± 0.65	71.79 ± 0.90	33.49 ± 1.34	25.89 ± 1.39	3572	24.70 ± 0.33
500	70.21 ± 1.16	65.90 ± 1.20	29.59 ± 1.44	25.08 ± 1.27	4387	20.70 ± 1.83
*q*_m_ (mg/g)	729.07 ± 12.08	645.96 ± 11.75	292.48 ± 14.19	252.80 ± 12.80		

Adsorption Desalination Isotherm Parameters					
isotherm model	parameters	Ca	Mg	K	Na
Langmuir isotherm	*q*_m (cal)_ (mg/g)	2245.09	1412.19	342.44	291.45
	*K*_L_ (L/g)	0.003	0.006	0.009	0.01
	*R*^2^	0.99	0.97	0.88	0.97
Freundlich isotherm	*K*_f_ (L/g)	11.59	16.39	15.05	18.99
	*n*	1.20	1.33	2.00	2.32
	*R*^2^	0.99	0.95	0.90	0.99

a Sorbents amount = 5 g; Sample feed volume = 20 mL; Seawater samples from Hatteras Beach, NC; Contact time 24 h.

The pristine sorbents exhibit higher AD efficiency (>70%) for Ca^2+^ and Mg^2+^ cations with less than 8% efficiency reduction at 500 ppm brine concentration. However, the sorbent’s AD efficiency for Na^+^ and K^+^ shows an almost half-fold reduction from the initial efficiency of ∼53% to 30% for K^+^ and 49 to 25% for Na^+^, respectively, suggesting sorbents high efficacy for remediating low-salinity water with moderate efficacy for cleansing alkali cations (Na^+^ and K^+^). The plots of individual cation’s AD efficiency with respect to the brine concentration (Figure S3) suggest that the AD efficiency of the pristine sorbents for Ca^2+^ and Mg^2+^ gradually decreases with the increase in concentration without reaching desalination equilibrium for the selected brine concentration range (Figure S3a,b). However, the AD efficiencies of Na^+^ and K^+^ gradually decrease with the increase of the brine concentration, and eventually reach a desalination equilibrium at 400 ppm, maintaining the AD efficiencies at 25 and 29% (Figure S3c,d). The results convey that adsorption sites of the sorbents for Na^+^ and K^+^ ions at higher concentrations reach partial saturation while removing the hardness of brine continually exceeding 65% in high-concentration brine. The total salinity removal efficiency of pristine sorbents decreases with respect to different salinity concentrations and reaches desalination equilibrium with a salinity removal efficiency of 20% at 4000 ppm, suggesting that sorbents could practically be utilized for the desalination of brine with widely varying concentrations.

The adsorption characteristics of the sorbent for each competing analyte in brine solutions were also evaluated by obtaining the adsorption isotherms for each analyte with a contact time of 24 h and are depicted in Figure 10c–f. The isotherms exhibit a steady increase in equilibrium adsorption capacities (*q*_e_) with respect to equilibrium concentrations (*C*_e_) and eventually reach adsorption equilibrium, yielding maximum average adsorption capacities of 729, 646, 292, and 253 mg/g for Ca^2+^, Mg^2+^, K^+^, and Na^+^, respectively (Table 4). The isotherms of all four cations follow the Langmuir and Freundlich isotherm models,^37,38^ implying the monolayer and multilayer surface adsorption, eventually reaching the saturation of sorbent’s surface-active sites. The adsorption isotherm parameters calculated for Langmuir and Freundlich isotherm models (eqs 1–4) are summarized in Table 4. The Langmuir maximum adsorption capacities calculated for Ca^2+^ and Mg^2+^ are significantly high, further convincing the sorbent’s suitability for removing the hardness of water. Agreeing with the Freundlich isotherm model, Fe(III)-TA exhibits multilayer adsorption for all cations but rather higher surface adsorption affinity for Ca^2+^ and Mg^2+^ ions, promoting heterogeneous adsorption.

**Figure 11:**
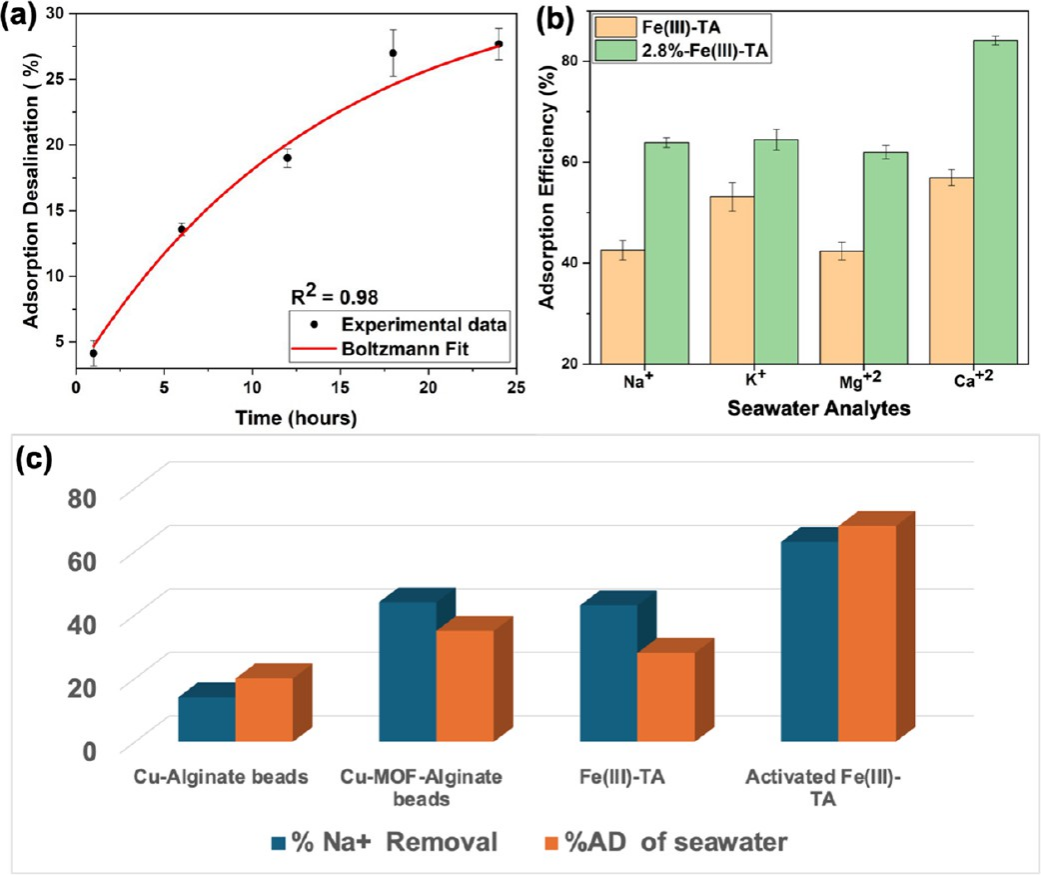
(a) Contact time vs adsorption desalination efficiency of seawater (Hatteras Beach, NC), using pristine Fe(III)-TA sorbents, (b) comparison adsorption desalination efficiencies of alkali and alkaline cations in seawater using pristine sorbent and activated sorbents (contact time 24 h), and (c) comparison plot for Na^+^ removal efficiency and AD efficiency of seawater using previously reported biobased sorbents with pristine and activated Fe(III)-TA sorbents.

Batch adsorption desalination studies on the field-collected seawater samples were also conducted using pristine sorbents and activated sorbents with 2.8%NH_4_OH. The adsorption desalination efficiency of the pristine sorbent with respect to different contact times was first evaluated and is depicted in Figure 11a. The pristine sorbents exhibit a gradual increase in adsorption efficiency with time and reach adsorption desalination equilibrium at 24 h. As summarized in Table 5, The adsorption desalination efficiency of the pristine sorbents for the seawater with an average salinity of 6315 ppm was found to be ∼28% at 24 h and is slightly higher than the salinity removal efficiency of brine with a concentration of 4000 ppm. Following the comparison plot of AD efficiency of pristine sorbent and activated sorbent for seawater desalination (Figure 11b), the pristine sorbents show lower adsorption efficiencies for alkali and alkaline cations, whereas activated sorbents have improved the AD efficiencies, with almost 20% increase in the efficiency. The activated sorbents exhibit the highest adsorption (84%) for Ca^2+^ and all other cations adsorb with an adsorption efficiency in the range of 61–63%. The amphoteric nature of the sorbent is attributed to the improved adsorption efficiency upon activation of the sorbent with 2.8%NH_4_OH, increasing negatively charged active sites on the sorbent’s surface by deprotonating residual hydroxyl groups within catechol units of the tannic acid.^32^ As we described in our recently published work, this tailored activation process of switching the surface charges from neutral to negatively charged surface, allows us to improve the adsorption affinity for cations over anions, yielding high removal efficiency of alkali and alkaline cations, thereby resulting in higher AD efficiency overall compared to pristine sorbents.^32^

**Table 5:** Summary of AD Efficiencies of Analytes in Seawater and Comparison of Na^+^ Removal and %AD of Seawater Using Pristine and Activated Fe(III)-TA Sorbents with Prior Reported Biobased Sorbents

adsorption efficiency of analytes in seawater (%)				previously reported sorbents	
analyte	concentration (ppm)	adsorption efficiency by pristine Fe(III)-TA (%)	adsorption efficiency by 2.8% NH_4_OH treated Fe(III)-TA (%)	adsorption efficiency by Cu-Alginate^75^ (%)	adsorption efficiency by Cu-MOF-Alginate^75^ (%)
Na	5153.27	42.59 ± 1.92	63.90 ± 0.95	14 ± 1.0	44 ± 1.0
Mg	503.48	42.36 ± 1.72	61.97 ± 1.35		
K	162.23	53.16 ± 2.83	64.45 ± 2.06		
Ca	452.29	56.94 ± 1.58	84.12 ± 0.87		
%AD of seawater		27 ± 1.18	68.61 ± 1.30	20 ± 1.0	35 ± 5.0

Comparative analysis was also conducted to evaluate the potential utilization of our biosorbent over similar biobased metal–organic framework-derived sorbents for efficient removal of Na^+^ ions and adsorption desalination of seawater. Thus, we compared our sorbent’s adsorption efficiency for Na^+^ ions and AD efficiency of seawater with Cu-Alginate beads and Cu-MOF-Alginate beads, which are the most structurally attributed sorbents for the comparison with Fe(III)-TA sorbents. As summarized in Table 5, in terms of Na^+^ removal and AD efficiency, our biobased sorbents exhibit higher performance compared to the sorbents of Cu-Alginate or Cu-MOF-Alginate while offering significant performance in removing other alkali and alkaline cations with high adsorption capacity.

### 2.4 Study on the Sorbent’s Disinfection Performance

**Figure 12:**
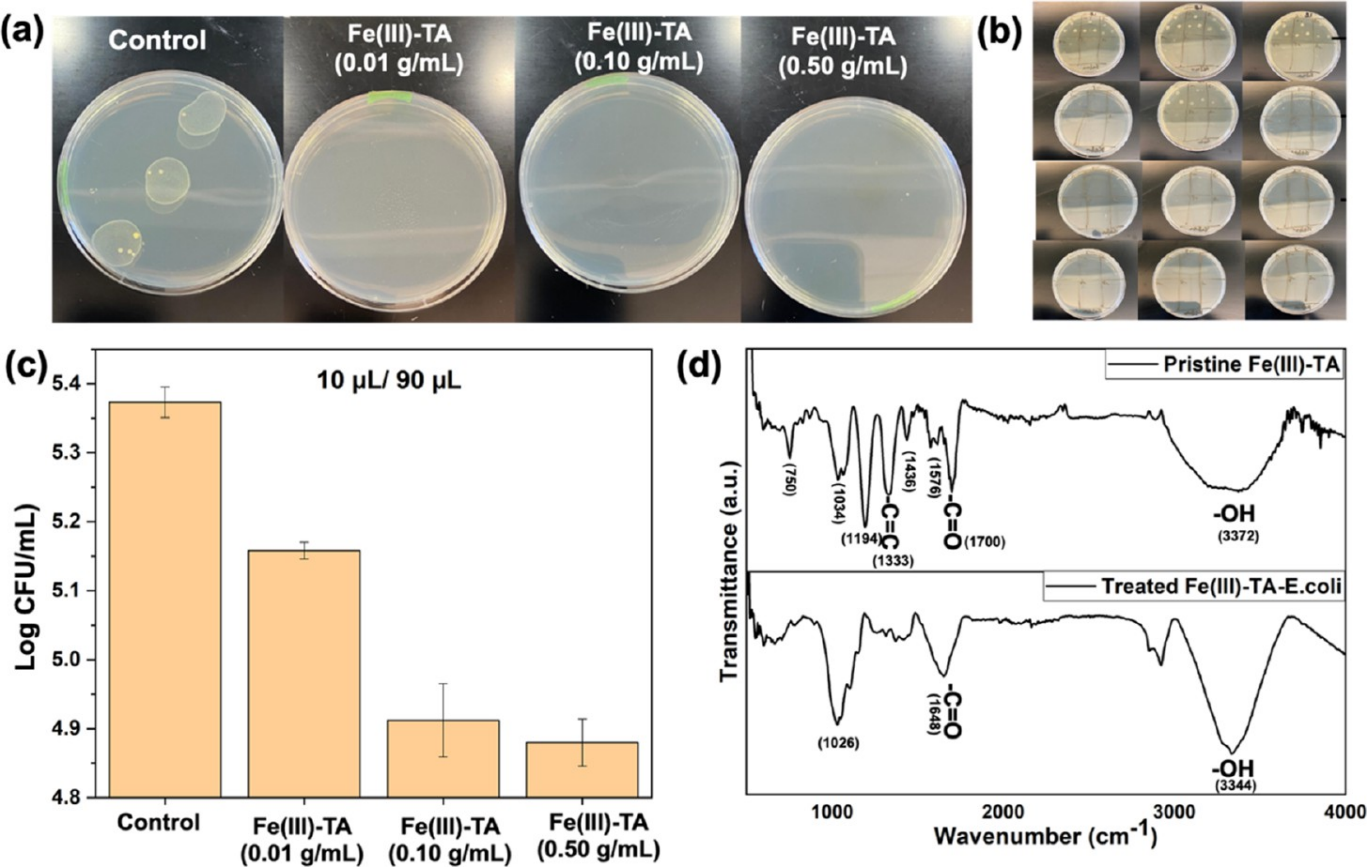
Board spectrum of antimicrobial activity for seawater disinfection: (a) Images of agar plates after incubation (at 35 °C) seawater samples treated with different doses of the sorbents and the culture plate of untreated seawater sample (control). (b) Images of agar plates with cultures used for CFU analysis, after treating the seawater culture solutions at different concentrations of the sorbent followed by incubation at 35 °C. (c) Plot of CFU assay analysis with respect to different concentrations of the sorbent. (d) Fourier transform infrared (FTIR) spectral traces of pristine Fe(III)-TA and treated Fe(III)-TA.

The sorbent’s ability to cleanse pathogens from seawater was studied with respect to the different doses of sorbents. In a typical disinfection experiment, seawater samples were treated with different doses of sorbents at a contact time of 15 min followed by an aliquot of treated seawater samples being drop-cast on agar plates. The agar plate samples, incubated at 35 °C for 90 h, are depicted in Figure 12a along with the control culture plate obtained for untreated seawater. Compared to the control, seawater samples treated with different doses of sorbents exhibit minimal microbial growth, conveying the antimicrobial properties of the sorbents. The colony formation unit (CFU) assay was conducted to quantify the efficacy of the sorbent’s antimicrobial performance by treating a series of culture solutions, obtained from the original culture plates of untreated seawater, at different doses of sorbents. The images of culture plates obtained from treated culture solutions along with the control are shown in Figure 12b. The images imply that the number of viable microbes is reduced in treated seawater, supporting the disinfection of seawater samples by the sorbents. The CFU assay analysis confirms the rapid removal of pathogens with a 67% disinfection efficiency for only 15 min contact time at the sorbent dose of 0.5 g/mL (Figure 12c).

The CFU analysis results discussed above confirm only the viable microbes present in the treated water but do not reveal the fate of the microbes adsorbed onto the sorbent. To prove that sorbents participate in the inactivation of microbes, following a contact mode mechanism, a sample of sorbents, used for disinfection of seawater was subjected to a well-plate system (Figure S4a) as described in the Experimental Section. The well plates were subjected to CFU analysis to determine the viability of the microbes adsorbed onto the sorbent. No viable colony growth was detected in the used sorbents sample, implying that all of the bacteria adsorbed onto the sorbents were inactivated. This verifies the active mode of disinfection by the sorbent’s surface.

The antimicrobial performance of Fe(III)-TA is significant in terms of the shorter contact time and the very low dose of the sorbent as our sorbent can remediate seawater in bulk volume by cleansing diverse microorganisms present in seawater, compared to known antimicrobial polymer gels^76−78^ and nanoparticles-impregnated biobased polymer beads.^79^ Supporting from the prior literature on the antimicrobial properties of tannic acid^80^ and iron(III)-tannate complexes,^81^ it is our thesis that catechol units of tannic acid and Fe(III) metal ion nodes participate in the antimicrobial activity by inactivating the live microbes adsorbed by the sorbent’s surface. To prove our thesis, we conducted FTIR and XPS analyses after treating the sorbents with seawater. As depicted in Figure 12d, the vibronic stretching for Fe–O at ν_(Fe–O)_ = 750 cm^–1^ in the treated sorbents is significantly diminished, compared to the respective vibronic stretching in pristine sorbent’s FTIR spectrum. This result indicates that metal ions were consumed by the microbes, eventually leading to cell death due to the metal ion cytotoxicity. Additionally, comparison to the pristine sorbent’s vibronic stretching of tannic acid, representing C–O, C=C, and C–O–C of catechol and pyrogallol groups at vibronic frequencies of 1194, 1333, 1436, and 1576 cm^–1^ are poorly resolved in the treated sorbents, suggesting that there are structural changes in tannic acid’s functional groups. These structural changes could be due to the participation of tannic acid’s catechol groups in the deactivation of microbes, acting as an antioxidant.^80,81^ The vibronic frequency shift of the ester carbonyl in pristine sorbent from ν_(C=O)_ of 1700 cm^–1^ to ν_(C=O)_ of 1648 cm^–1^ in treated sorbent is an indication of the chemical interaction changes in the carbonyl groups. It is possible that the shift in carbonyl stretching and the diminished vibronic stretching of aromatic C=C and C–O bonds could be attributed to the oxidation of catechol units to semiquinone/quinone form, evidencing the antioxidant activity of tannic acid.

**Figure 13:**
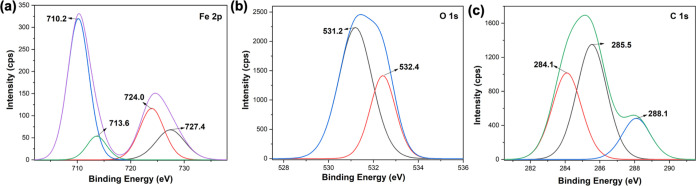
Binding energy spectra of (a) Fe 2p, (b) O 1s, and (c) O C 1s obtained for the treated sorbents.

The elemental composition analysis (Table 6) and the binding energy spectra of Fe 2p, C 1s, and O 1s, obtained for the treated sorbents, support the FTIR spectral results (Figure 13). The elemental composition analysis shows a half-fold reduction of iron content in the treated sorbent compared to the pristine sorbents (Table 6), confirming that metal ions were consumed by the microbes. Additionally, the carbon and oxygen contents were also decreased ∼14 and ∼20%, respectively, in the treated sorbents compared to the %weight of carbon and oxygen in the pristine sorbents. The reduction in the elemental composition of Fe^3+^, C, and O further conveys the involvement of metal ion nodes and tannic acid in the microbes’ deactivation process. The binding energy spectrum of Fe 2p obtained for the treated sorbent (Figure 13a) exhibits spectral changes with considerable binding energy shifts, especially, corresponding to the satellite peaks of 2p_1/2_ /Fe^2+^ and 2p_1/2_ /Fe^3+^ at 724.8 and 727.4 eV in treated sorbents. These spectral attributes support the changes in the chemical environment of the metal oxide nodes, compared to the respective binding energy peaks of the Fe 2p binding energy spectrum for the pristine sorbent (Figure 7e). The O 1s binding energy spectrum (Figure 13b) of treated sorbent shows only a broader peak with a slight shoulder, which corresponds to the binding energy peaks for C–O (sp^3^) at 531.2 eV and C=O (sp^2^) at 532.4 eV. However, the O 1s binding energy spectrum of pristine sorbent (Figure 7m) shows a rather well-resolved spectrum, with a binding energy peak at 529.6 eV for Fe–O and a much-resolved shoulder peak at 532.4 eV for C=O. This further suggests that treated sorbents’ metal oxide nodes could be no longer in the form of Fe–O and the chemical environment of carbonyls has also changed. The binding energy spectrum of C 1s obtained for the treated sorbents also exhibits some spectral differences, with a binding energy shift for C–O and a well-resolved shoulder peak for C=O at 288.1 eV, instead of one broader binding energy spectrum (Figure 7i) for the pristine sorbent, providing definitive conformation for the chemical changes in tannic acid upon microbes’ interactions followed by deactivation.

**Table 6:** Elemental Composition and Binding Energies of Pristine and Treated Fe(III)-TA

Pristine Fe(III)-TA			
element type	%elemental composition	binding energies (eV)	bonding type/oxidation state
C 1s	47.25	284.1, 286.7, 288.1	C–C (*sp*^3^), O–C=O ()
O 1s	41.05	529.6, 531.0 (broad), 532.4	Fe–O, C–O (sp^3^), C=O (sp^2^)
Fe 2p	11.70	709.8, 714.4, 722.8, 726.7	2p_3/2_/Fe^3+^, 2p_3/2_/Fe^3+^ (Satellite Peak), 2p_1/2_/Fe^2+^, 2p_1/2_/Fe^3+^

Treated Fe(III)-TA			
element type	%elemental composition	binding energies (eV)	bonding type/oxidation state
C 1s	33.22	284.1, 285.5, 288.1	C–C (*sp*^3^), O–C=O ()
O 1s	21.71	531.2, 532.4	C–O (*sp*^3^), C=O (sp^2^)
Fe 2p	5.39	710.2, 713.6, 724.8, 727.4	2p_3/2_/Fe^3+^, 2p_3/2_/Fe^3+^ (Satellite Peak), 2p_1/2_/Fe^2+^, 2p_1/2_/Fe^3+^

**Figure 14:**
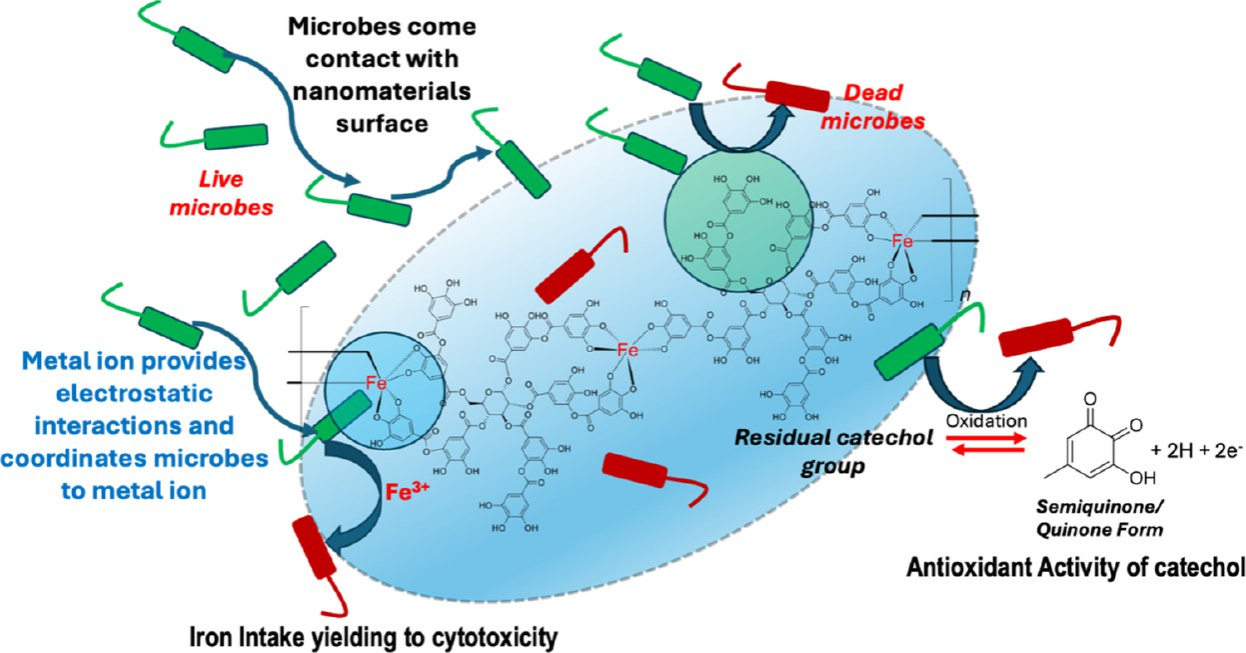
Schematic diagram for the postulated contact active mode disinfection mechanism of Fe(III)-TA, representing cell death by cytotoxicity of iron cations and antioxidant activity of catechol, forming semiquinone and quinone reactive species.

Aligning with the FTIR spectral changes, XPS results evidence that iron cations in metal oxide nodes and catechol units of tannic acid participate in microbes’ deactivation via (1) scavenging iron cations by the microbes from the metal oxide nodes and (2) partial oxidation of catechol units to semiquinones and quinones, exhibiting antioxidant activity, thereby leading to cell death. Thus, we could postulate that disinfection occurs via a contact active mode of inactivation, causing cell death due to the cytotoxicity from iron intake and the antioxidant activity of catechol units in tannic acid. As illustrated in Figure 14, we propose the following mechanism, which follows a dual-mode deactivation process by Fe^3+^ of metal oxide nodes and catechol units of the coordination polymer framework. The cytotoxicity from metal ions and reactive quinone species results in cell death. Our future studies will focus on understanding the oxidation of catechol units to quinone derivatives during the microbes’ deactivation process and the role of metal ion nodes of the sorbent in the formation of quinone derivatives.

## 3 Conclusions

The biosorbent Fe(III)-tannate demonstrates the proof-of-feasibility for heavy-metal treatment, adsorption desalination, and disinfection. The sorbents exhibit outstanding adsorption capacities for Ag^+^, Cd^2+^, and Pb^2+^ with the maximum experimental adsorption capacities (*q*_m_) of 96.25, 66.54, and 133.83 mg/g at neutral pH, respectively. The adsorption equilibrium isotherms of heavy metals follow the Langmuir and Freundlich isotherm models, conveying the monolayer adsorption for the full concentration range of 10 ppm to 500 ppm and multilayer adsorption for the low concentration range of <50 ppm. Deviating from the linear form of the Temkin isotherm model, the sorbent exhibits a sigmoidal adsorption isotherm (S_II_-type), favoring the heavy metals’ dependence on the surface adsorption at room temperature followed by micropore filling of analytes. The adsorption kinetic isotherm models, pseudo-first-order, pseudo-second-order, and Elovich isotherms convey the kinetic behavior and rates of analytes adsorption, supporting physisorption and chemisorption of heavy-metal ions onto the sorbent’s surface. Agreeing with the adsorption equilibrium isotherms and adsorption kinetic isotherms, XPS and TEM/EDS results have provided additional evidence to postulate the sorbent’s heavy metals adsorption mechanism. The stepwise adsorption process follows the formation of fluid films, facilitating the surface adsorption via physisorption, external mass transfer diffusion, followed by chemisorption, and the subsequent internal mass transfer process, filling the sorbent’s pores. The adsorption desalination studies confirm the effective removal of salinity while cleansing alkali and alkaline cations present in brine and seawater. The adsorption isotherms of the sorbents for alkali and alkaline cations present in brine solutions follow Langmuir and Freundlich isotherm models, yielding maximum average adsorption capacities of 729, 646, 292, and 253 mg/g for Ca^2+^, Mg^2+^, K^+^, and Na^+^, respectively. The results obtained from different concentrations of synthetic brine and seawater samples provide a direct assertion for the sorbent’s effectiveness over a wide range of salinity concentrations of water resources. The activated sorbents exhibit improved desalination efficacy compared to the efficiency of pristine sorbents, while both activated and pristine sorbents show high affinity for Ca^2+^ and Mg^2+^. The ability to disinfect a wide range of microbes in seawater with 67% disinfection efficacy is an important intrinsic property of this novel biosorbent for point-of-use water disinfection applications. The postulated disinfection mechanism follows deactivation of microbes due to the cytotoxicity of iron cations and the antioxidation activity of tannic acid, yielding reactive quinone species.

The capability to treat heavy metals, desalinate dissolved salts, and disinfect by one type of biosorbent, with multiplex function, is a unique trait for a stand-alone high-performance filter unit to apply for the tertiary treatment of water. Nonetheless, the challenges affecting the efficiency, cost-effectiveness, and environmental sustainability of water remediation processes include: (1) energy intensity, especially prevalent in reverse osmosis and membrane technologies, (2) the cost of implementation, (3) scale and infrastructure, (4) waste and sludge accumulation, and (5) environmental impact. In turn, this *3-in-1* innovative biosorbent is an efficient, point-of-use, and self-contained solid-phase adsorption-driven remediation technology that operates with minimal energy consumption, low-cost, zero carbon emissions, and no discharge of brine. Additionally, this approach could tie into the concept of circular economy by utilizing existing resources effectively to improve water quality and quantity, enabling a decentralized surface and groundwater remediation unit for irrigation purposes, while employing climate-smart agricultural practices and leading to an innovative solution for global freshwater scarcity.

## 4 Experimental Section

### 4.1 Materials

Tannic acid (C_76_H_52_O_46_) (IUPAC Name: 1,2,3,4,6-penta-*O*-{3,4-dihydroxy-5-[(3,4,5-trihydroxybenzoyl)oxy]benzoyl}-d-glucopyranose) (molar mass = 1701.19 g mol^–1^), iron(II)acetate hexahydrate, cadmium(II) acetate (Cd (OCOCH_3_)_2_, molar mass = 230.5 g mol^–1^), and anhydrous ethanol (200 proof) were obtained from Sigma-Aldrich. Silver nitrate (AgNO_3_, molar mass = 169.87 g mol^–1^), lead acetate (Pb (OCOCH_3_)_2_, molar mass = 325.29 g mol^–1^), sodium chloride (NaCl, molar mass = 58.44 g mol^–1^), and magnesium chloride hexahydrate (MgCl_2_·6H_2_O, molar mass = 230.30 g mol^–1^) were obtained from VWR Chemicals. Calcium chloride dihydrate (CaCl_2_·2H_2_O, molar mass = 147.01 g mol^–1^) and potassium carbonate (K_2_CO_3_, molar mass = 138.205 g mol^–1^) were obtained from Acros Organics. Multielement standard was purchased from SPEX CertiPrep. Unless otherwise stated, all chemicals were used as received. Seawater samples were collected from Hatteras Beach, NC.

### 4.2 Characterization

The chemical composition and functional groups were analyzed using Fourier transform infrared spectroscopy (FTIR-Varian 670-IR spectrometer). The morphology and the electron diffraction images of pristine sorbents and heavy-metal-ion-adsorbed sorbents, along with their respective elemental mapping, were obtained from transmission electron microscopy (HR-TEM JEOL2100PLUS with STEM/EDS capability at 120 and 200 kV, respectively) coupled with electron diffraction spectroscopy (EDS). The oxidation states of each element were obtained by X-ray photon spectroscopy (XPS-Escalab Xi+-Thermo Scientific). Elemental compositions for Fe, C, and O were also obtained from XPS elemental survey analysis. Detection of heavy-metal ions and other alkali and alkaline cations in synthetic brine solutions and seawater samples before and after soaking with sorbents was conducted using a simultaneous inductively coupled plasma optical emission spectrometer (Varian 710-ES ICP Spectrophotometer), equipped with trace element analysis capability, spanning wavelengths from 177 to 785 nm. Sample preparation methods for inductively coupled plasma optical emission spectrometer (ICP-OES) analysis are described in the Supporting Information.

### 4.3 Equilibrium Adsorption Isotherm Studies

The adsorption capacity of heavy metals Pb^2+^, Cd^2+^, and Ag^+^ onto Fe(III)-TA adsorbents was studied by using a series of experiments at varied initial heavy-metal-ion concentrations ranging from 10 to 500 ppm. Additionally, experiments were conducted with lower concentrations of heavy metals, specifically at 0.1 and 1.0 ppm. In each experiment, adsorbents (20.0 mg) were accurately measured and transferred into glass vials. From each heavy-metal-ion solution, a volume of 10.0 mL was added to each glass vial. The glass vials were sealed, and the suspension was sonicated for 5 min and subsequently left to sit undisturbed for 15 min. After the stipulated time, the treated solutions were recovered.

### 4.4 Kinetic Adsorption Isotherm Studies

The effect of contact time on the adsorption capacity of Fe(III)-TA for three heavy-metal ions was studied using respective heavy-metal solutions with a concentration of 500 ppm and sorbent amount of 20.0 mg. In a typical experiment, to a vial charged with the sorbents (20.0 mg) was added a heavy-metal solution (10.0 mL), and the mixture was sonicated for 5 min and left to sit undisturbed over 30 min while taking an aliquot (100 μL) over the intervals of 5, 10, 15, 20, 25, and 30 min.

### 4.5 pH-Dependent Adsorption Isotherm Studies

The effect of solution pH on the adsorption capacity for three heavy metals by Fe(III)-TA adsorbent was studied by adjusting the heavy-metal-ion solutions’ pH range from 2 to 9. For this analysis, heavy-metal solutions (10.0 mL) with a concentration of 500 ppm were used. For each solution, pH was adjusted using 0.1 M HCl or 0.05 M NaOH. The pH-adjusted heavy-metal solutions prepared in this manner were soaked undisturbed over 30 min with the sorbents (20.0 mg), after sonicating for 5 min.

### 4.6 Temperature-Dependent Adsorption Studies

The effect of solution temperature on the adsorption efficiency of a Pb^2+^ by Fe(III)-TA was studied by maintaining the temperature of the sorbents-charged Pb^2+^ solutions from 25 to 55 °C with an increment of 10 °C. The heavy-metal ion solutions with a concentration of 100.0 ppm were prepared by dissolving lead(II) acetate (39.25 mg) in deionized (DI) water (50 mL). Subsequently, 10.0 mL of this solution was distributed into four separate vials. The vials were sealed with caps and subjected to thermal conditions of 25, 35, 45, and 55 °C using hot plates. Temperature stabilization was verified with an infrared thermometer. Following temperature equilibration, 20.0 mg of Fe(III)-TA sorbents were introduced into each Pb^2+^ solution and allowed to equilibrate undisturbed for 15 min at each controlled temperature. The resulting suspensions were then filtered through a 0.45 μm filter for ICP-OES analysis.

### 4.7 Adsorption Desalination Studies Using Synthetic Brine and Field-Collected Seawater Samples

Synthetic brine solutions were prepared by dissolving alkali (NaCl and K_2_CO_3_) and alkaline (MgCl_2_·6H_2_O and CaCl_2_·6H_2_O) salts in DI water, maintaining the ratio between each cation at 1:1 and the initial concentrations ranged from 100 to 500 ppm. In a typical procedure for synthetic salinity water samples, salts of respective cations with relevant weights (NaCl—0.254 g, 0.508 g, 0.762 g, 1.016 g, and 1.271 g; MgCl_2_6H_2_O—0.837 g, 1.673 g, 2.509 g, 3.345 g, and 4.183 g; CaCl_2_2H_2_O—0.367 g, 0.734 g, 1.1 g, 1.467 g, and 1.834 g; and K_2_CO_3_—0.177 g, 0.353 g, 0.529 g, 0.705 g, 0.883 g) were dissolved in deionized water (1 L). Adsorption equilibrium analyses were conducted using a fixed-bed batch adsorption process, employing a fitted-disc glass column (inner diameter: 1 in., length: 10 in.) filled with pristine Fe(III)-TA sorbents (5.0 g). The column was filled with dry granular sorbents (granular size ranged from ∼1 to 3 mm), yielding a loosely packed sorbent bed of ∼1 in. height. The synthetic solutions (10.0 mL) were added slowly through the wall of the glass column, without disturbing the sorbent layer, and the columns were left undisturbed over 24 h prior to take out for ICP analysis using 3% v/v nitric acid.

### 4.8 Procedure for %AD Efficiency Analysis in Seawater

The adsorption desalination efficiency of seawater samples collected from Hatteras Beach, NC, was also studied using fixed-bed batch adsorption. In a typical procedure, seawater was first filtered through a microfilter (0.45 μm) to remove any solid particles. A volume of filtered seawater (20.0 mL) was added to a glass beaker (50.0 mL) and charged with pristine sorbents (5.0 g). The TDS readings were taken by immersing the digital TDS meter probe into the suspension at different time intervals (1, 6, 12, 18, and 24 h). The TDS meter probe was thoroughly rinsed in DI water between each measurement.

### 4.9 Adsorption Efficiency Analysis of Alkali and Alkaline Cations in Seawater

The adsorption efficiency of each analyte in seawater was also studied. A volume of filtered seawater (10.0 mL) was added to the glass column and charged with the pristine sorbent (5.0 g). After 24 h, aliquots of seawater samples were collected from the column and subjected to ICP-OES analysis using 3%v/v nitric acid. This study was repeated, using activated sorbents using 2.8% NH_4_OH. In a typical activation process, the adsorbents (10.0 g) were soaked in the prepared 2.8% NH_4_OH (20.0 mL) sealed with paraffin wax and left to soak for 24 h undisturbed. The activated sorbents were recovered through vacuum filtration and dried under a hood overnight. The pH of the activated Fe(III)-TA adsorbents, using 2.8% NH_4_OH solutions, in DI water was measured to be 7.58, compared to the pH of nonactivated sorbents (pH = 4.93) in water. The activated sorbents (5.0 g) were used for the fixed-bed column and soaked with seawater (10.0 mL) over 24 h, and the eluent was analyzed using ICP-OES.

## Supporting Information Available

Isotherm plots for heavy metals and adsorption efficiency plots for alkali and alkaline cations with respect to different brine concentrations, experimental methods of the sample preparation for the ICP-OES analysis, detailed description of heavy-metal adsorption capacity analysis, experimental methods for UV–vis spectroscopy analysis, colony formation unit assay analysis, and the methods for pathogens disinfection analysis (PDF)
