# Supplementary material for: Insight into Iron(III)-Tannate Biosorbent for Adsorption Desalination and Tertiary Treatment of Water Resources
Source: ACS Omega. 2024 Dec 19;10(1):239–60. doi: 10.1021/acsomega.4c05152 (PMC11739983; doi:10.1021/acsomega.4c05152)
Supplement: Supplementary file 1 — ao4c05152_si_001.pdf [file ao4c05152_si_001.pdf]

Supporting Information

Insight to Iron(III)-Tannate BioSorbent for Adsorption Desalination and Tertiary Treatment of Water Resources

*Kelvin Adrah, Gayani Pathiraja, and Hemali Rathnayake**

Nanoscience Department, University of North Carolina Greensboro, Greensboro, NC, 27401, USA.

**Correspondence: hprathna@uncg.edu; Tel.: 01-336-285-2860.*

Table S1: Comparison summary table of adsorption capacities of Fe(III)-TA with previously published natural sorbents for heavy metals of Ag^{+}, Cd^{2+}, and Pb^{2+}

**Sorbent**	**Heavy metal ions qmax (mg/g)**			**Reference**
	**Ag^{+}**	**Cd^{2+}**	**Pb^{2+}**	
Chitosan	38.46	-	-	[41]
Chitosan/montmorillonite	43.48	-	-	
Chitosan/bamboo charcoal composites	52.91	-	-	[42]
Modified lignin	-	6.70–7.50	8.20–9.00	[43]
Porous lignin-based sphere	-	-	14.90	[45]
Cell-ethylenediaminetetraacetic acid	-	33.20	41.20	[49]
Cell-carboxymethyl	-	23.00	63.40	
MMSCB 3	-	86.20	158.70	[50]
MMSCB 5	-	106.40	222.20	
Fe(III)-TA	68.41	43.08	147.66	Present study

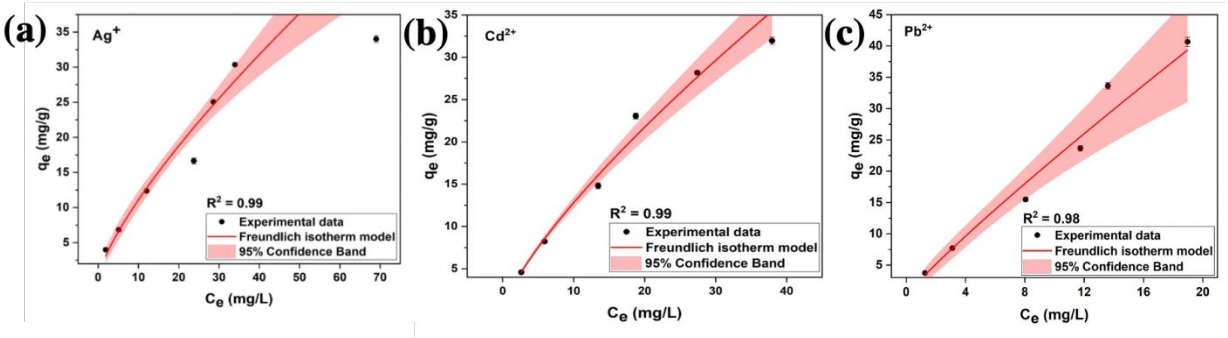

Figure S1: Freundlich adsorption isotherm plots for: (a) Ag^+, (b) Cd^{2+}, (c) Pb^{2+} at low adsorbate concentration range (<50 ppm).

Table S2: Adsorption efficiency of Ag+, Cd2+, and Pb2+ onto Fe(III)-TA sorbents at heavy metal ion concentration of 0.1 ppm and 1 ppm.

**Heavy metal**	**Initial concentration (ppb)**	**Adsorption Efficiency (%)**
**Ag^+**	100.503	99.17±0.66
	1000.083	90.73±0.19
**Cd^{2+}**	107.976	92.78±0.33
	998.635	90.07±0.09
**Pb^{2+}**	98.725	99.02±0.14
	986.287	91.12±0.49

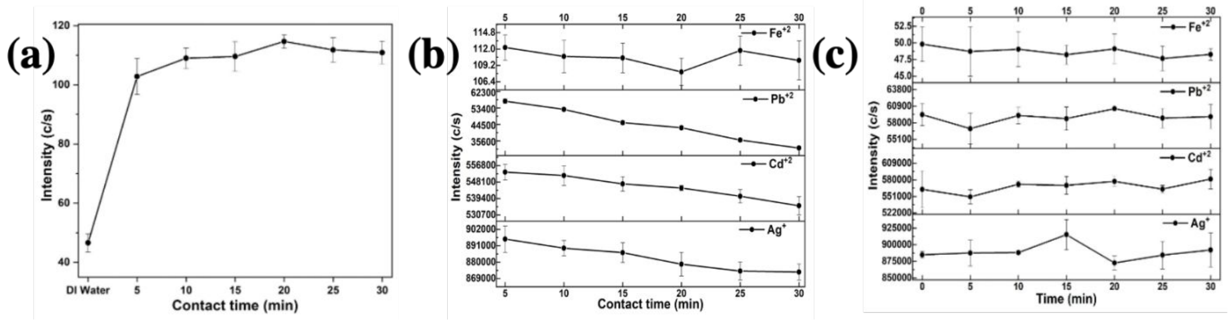

Figure S2: Intensities of Fe^{2+}, Ag^{+}, Cd^{2+}, and Pb^{2+} measured: (a) pristine Fe(III)-TA sorbents dispersed in DI water, (b) pristine Fe(III)-TA sorbents dispersed in heavy metal solution and (c) heavy metal solution

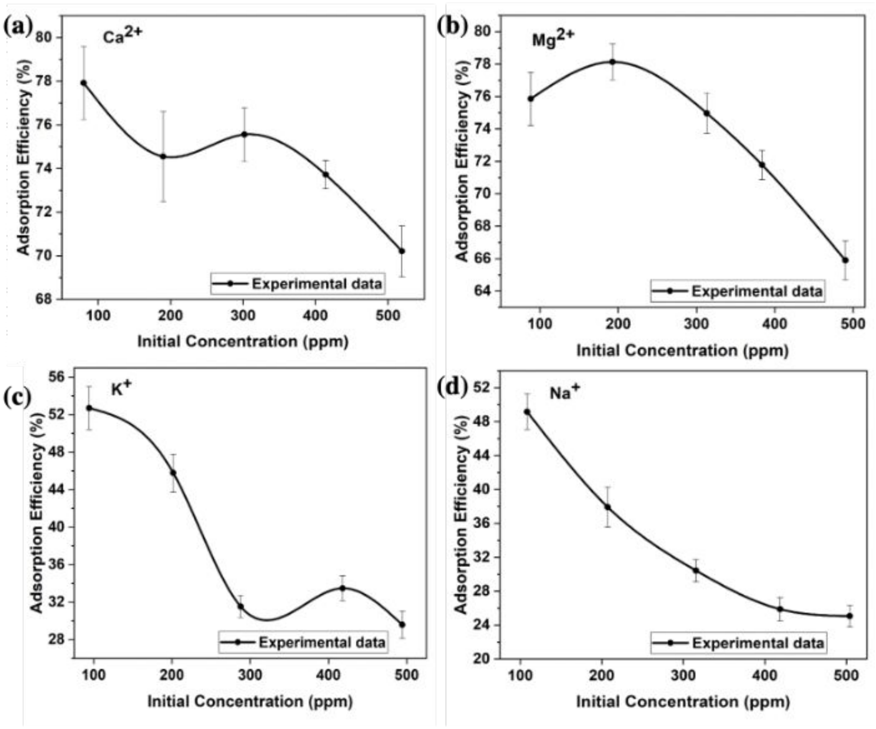

Figure S3: Adsorption efficiency of: (a) Ca^{2+}, (b) Mg^{2+}, (c) K^{+}, and (d) Na^{+} with respective to initial concentration in brine (100-500 ppm).

=

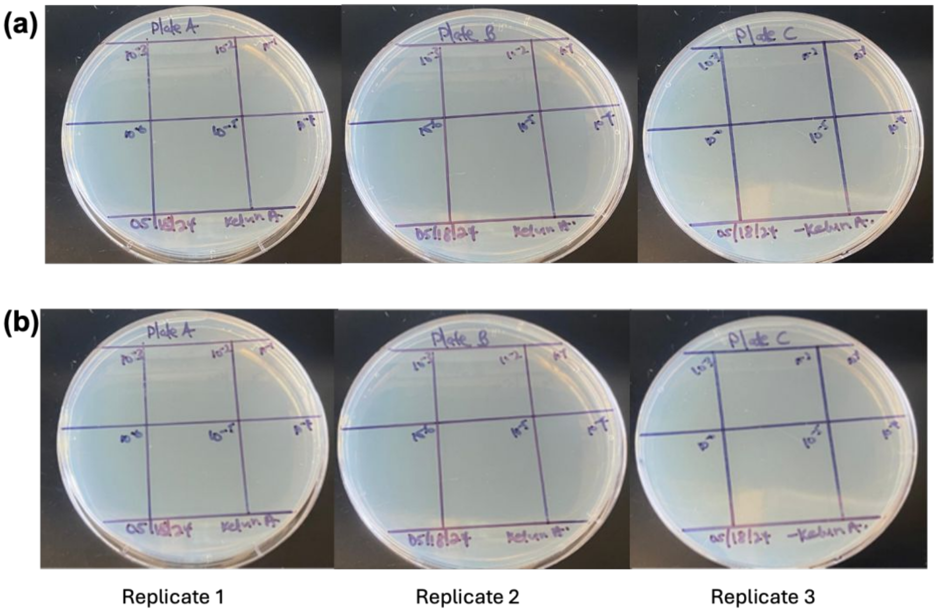

Figure S4: Culture plates with used sorbents after treated for seawater disinfection: (a) before incubation, and (b) after 24 hrs of incubation.

Experimental Methods

Preparation of heavy metal-ion stock solutions for adsorption equilibrium and kinetic isotherm studies: A set of stock solutions with the concentration 1000 ppm of Pb^{2+}, Ag^{+}, and Cd^{2+} was prepared by dissolving lead(II)acetate (1.56 g), silver nitrate (1.57 g), and cadmium(II)acetatedihydrate (1.10 mg) in DI water (50.0 mL), respectively. The stock solutions prepared in this manner were diluted with DI water to make a series of concentrations ranging from 10 ppm to 500 ppm for the adsorption equilibrium and kinetic isotherm studies.

Preparation of heavy metal standards for the ICP-OES calibration: Standard samples ranging from 10 ppm to 100 ppm lead, silver, and cadmium ions were prepared from each heavy metal ICP standards with the concentration of 1000 ppm.

Sample preparation for ICP-OES analysis: All eluents collected from adsorption equilibrium, kinetic, and pH studies were analyzed using ICP-OES in 3% (v/v) nitric acid solutions along with the initial heavy metal ions solutions and the heavy metal ions standards. All measurements were taken in triplicate. In a typical sample preparation procedure for ICP analysis, the eluents (initial and after treated with sorbents) were first filtered through a 0.45 µm filter. Following filtration, the eluents with concentration >100 ppm were diluted to a lower concentration (<100 ppm) by adding 3% (v/v) nitric acid solution. To obtain the calibration curve for each heavy metal ion, the standards with concentration of 100 ppm of cadmium, silver, and lead were prepared by adding 1.0 mL of each heavy metal ion standard to 9.0 mL of 3% (v/v) nitric acid solution. Standards prepared in this manner were diluted to make series of lower concentration standard solutions ranging from 10 ppm to 100 ppm by mixing with 3% (v/v) nitric acid solutions. From the calibration curve, initial and final heavy metal ions concentrations were obtained and used for calculating the experimental equilibrium adsorption capacities as follows.

Determining heavy metal ions content (experimental adsorption capacities): The amount of heavy metal ions adsorbed onto the sorbent at equilibrium (qe) was calculated by mass balance equation of heavy metal ions before and after the sorption using the equation (18) shown below.

$$q_e = \frac{(C_o - C_e)V}{W} \text{-----------(18)}$$

where q_e is the equilibrium adsorption capacity, C_o and C_e are initial and final heavy metal concentration (ppm), respectively at equilibrium, obtained from the ICP-OES analysis.

Sample preparation for UV-visible spectroscopy analysis: To study the interactions of heavy metal ions with the sorbent, UV-vis analysis was conducted for heavy metals adsorbed sorbents by redispersing in DI water. For this experiment, Fe(III)-TA adsorbents (100 mg) were dispersed three heavy metal ions stock solutions (50.0 mL of 1000 ppm solutions). The suspension was sonicated

for 5 minutes and allowed to settle undisturbed for 30 minutes. Subsequently, the adsorbents were recovered, dried in an oven at 60 °C for 1 hour, and allowed to cool at room temperature. Each heavy metal ion adsorbed sample (20.0 mg) was re-dispersed in DI water (5.0 mL) and sonicated for 5 minutes. From each suspension prepared in this manner, an aliquot (1.0 mL) was transferred into a cuvette for UV-visible analysis. These same suspensions were used to prepare the grids for HR-TEM analysis. An aliquot of sample was drop-casted on to a carbon-coated copper grid and air dried under ambient conditions over 24 hrs before imaging.

Colony formation unit (CFU) assay analysis: At three different adsorbent dosage (0.1, 1.0, and 5.0 g), the efficiency of sorbents disinfection was evaluated using seawater samples by conducting colony formation unit (CFU) assay for sorbents treated and untreated seawater samples. For CFU assays, pre-sterilize two agar plates were treated with treated (seawater samples soaked in different dosage of sorbents) and untreated seawater samples (50 μL) and air-dried in a sterile hood for 45 minutes. The agar plates were then incubated at 37 °C for 96 hours. The bacterial colonies were only observed from the agar plate with untreated seawater (control plate). The resulting bacterial colonies from the control plates were harvested using an inoculation loop and transferred into 30.0 mL of culture medium. The inoculum was introduced into a shaking incubator set to operate at 30°C and 130 rpm. Following a 24-hour incubation period, the culture was retrieved, and its optical density was measured at 600 nm, and adjusted the optical density from 0.3 to 0.17 with fresh media. Utilizing a sterile pipette, the culture (10.0 mL) was transferred into four conical flasks. To each conical flask Fe(III)-TA adsorbents (0.1, 1.0, and 5.0 g) were introduced along with a control. The conical flasks were kept in the shaking incubator, operating at 30°C while rotating at the rotation speed of 130 rpm. After an additional 3-hour incubation, the culture was retrieved. Following these incubation steps, serial dilutions of both the control and treatment cultures,

spanning dilution factors from 10^{-1} to 10^{-8} were prepared and dispensed into a 96-well plate. Subsequently, these dilutions (5.0 μL) were plated onto agar plates and subsequently incubated at 37°C for 24 hours. The colony forming unit/mL is calculated using the equation (19).

$$CFU = \frac{Number\ of\ colonies\ x\ total\ dilution\ factor}{Volume\ of\ culture\ plates\ (mL)} \text{-------(19)}$$

The mode of pathogens disinfection studies: Sorbents (1.0 g) which was used for 30 min treatment of seawater were transferred into a conical flask, containing fresh media (10 mL). The setup was allowed to remain undisturbed for 30 minutes to enable suspended sorbents to settle. Subsequently, the optical density, measured at 600 nm, was adjusted from 0.94 to 0.20 using fresh media. The conical flask was then placed in a shaking incubator set to operate at 30°C while rotating at 130 rpm. Following a 3-hour incubation period, the culture media was collected. Serial dilutions ranging from 10^{-1} to 10^{-8} were prepared and dispensed into a 96-well plate. These dilutions (5.0 μL each) were then plated onto agar plates and subsequently incubated at 37°C for 24 hours. The cultures were then subjected to the same CFU analysis procedure. This assay was repeated in three independent experiments.

Appendix

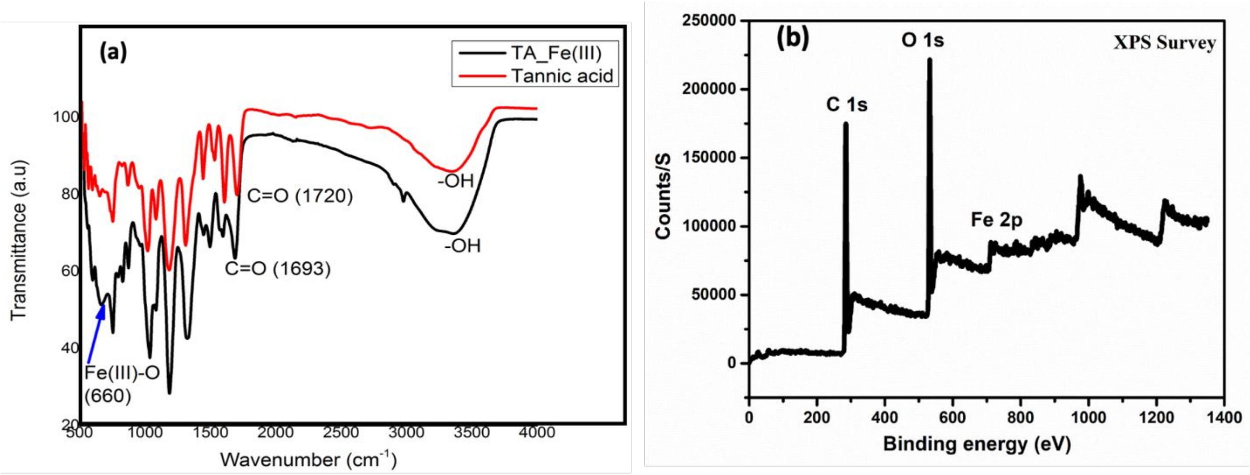

Figure S5: (a) FTIR spectra of Fe(III)-TA sorbents and tannic acid, and (b) XPS survey spectrum of Fe(III)-TA sorbents.

Table S2: Elemental composition and the binding energies of pristine Fe(III)-TA

Element type	%Elemental composition		Binding energies (eV)	Bonding type/oxidation state
	Experimental	Theoretical		
C 1s	47.25	46.28	284.1, 286.7, 288.1	C-C (*sp*^{3}), O-C=O (*sp*^{2})
O 1s	41.05	39.39	529.6, 531.0, 532.4	Fe-O, C-O (sp^3), C=O (sp^2)
Fe 2p	11.70	11.96	709.8, 714.4, 722.8, 726.7	$2p_{3/2}$ /Fe^{+3}, $2p_{3/2}$ /Fe^{+3} (Satellite Peak), $2p_{1/2}$ /Fe^{+2}, $2p_{1/2}$ /Fe^{+3}
